# Genomic Analysis, Evolution and Characterization of E3 Ubiquitin Protein Ligase (TRIM) Gene Family in Common Carp (*Cyprinus carpio*)

**DOI:** 10.3390/genes14030667

**Published:** 2023-03-07

**Authors:** Muhammad Aizaz, Yusra Sajid Kiani, Maryum Nisar, Shijuan Shan, Rehan Zafar Paracha, Guiwen Yang

**Affiliations:** 1 Shandong Provincial Key Laboratory of Animal Resistance Biology, College of Life Sciences, Shandong Normal University, Jinan 250061, China; 2 School of Interdisciplinary Engineering & Sciences (SINES), National University of Sciences and Technology (NUST), Islamabad 44000, Pakistan

**Keywords:** tripartite motif, common carp, ubiquitylation, phylogenetic tree, gene ontology

## Abstract

**Simple Summary:**

TRIM family proteins form a large family of proteins that play a central role in antiviral host defense. These proteins are characterized and identified in tetrapods but the knowledge related to this family among teleost species is very limited. In this study, we analyze the evolutionary relationships, characterization, and functional annotation of common carp TRIM family. The evolutionary analysis revealed that the TRIM family among tetrapods and teleost species is conserved in sequence similarity and domain architectures. The occurrence of certain motifs across the TRIMs members implies a conserved nature and indicate a group specific function. However, the exon-intron organization is more diverse suggesting the complex alternative splicing and duplication. The functional annotation shows that most TRIM proteins are associated with ubiquitination, which can potentially mediate multicellular processes including immune response, cell cycle regulation, modulation of different substrates of important signaling pathways and post translational modifications. The study results will open up new avenues for future research and can be used to elucidate several mechanisms related to common carp development and immune response.

**Abstract:**

Tripartite motifs (TRIM) is a large family of E3 ubiquitin ligases that play an important role in ubiquitylation. TRIM proteins regulate a wide range of biological processes from cellular response to viral infection and are implicated in various pathologies, from Mendelian disease to cancer. Although the TRIM family has been identified and characterized in tetrapods, but the knowledge about common carp and other teleost species is limited. The genes and proteins in the TRIM family of common carp were analyzed for evolutionary relationships, characterization, and functional annotation. Phylogenetic analysis was used to elucidate the evolutionary relationship of TRIM protein among teleost and higher vertebrate species. The results show that the TRIM orthologs of highly distant vertebrates have conserved sequences and domain architectures. The pairwise distance was calculated among teleost species of TRIMs, and the result exhibits very few mismatches at aligned position thus, indicating that the members are not distant from each other. Furthermore, TRIM family of common carp clustered into six groups on the basis of phylogenetic analysis. Additionally, the analysis revealed conserved motifs and functional domains in the subfamily members. The difference in functional domains and motifs is attributed to the evolution of these groups from different ancestors, thus validating the accuracy of clusters in the phylogenetic tree. However, the intron-exon organization is not precisely similar, which suggests duplication of genes and complex alternative splicing. The percentage of secondary structural elements is comparable for members of the same group, but the tertiary conformation is varied and dominated by coiled-coil segments required for catalytic activity. Gene ontology analysis revealed that these proteins are mainly associated with the catalytic activity of ubiquitination, immune system, zinc ion binding, positive regulation of transcription, ligase activity, and cell cycle regulation. Moreover, the biological pathway analyses identified four KEGG and 22 Reactome pathways. The predicted pathways correspond to functional domains, and gene ontology which proposes that proteins with similar structures might perform the same functions.

## 1. Introduction

The tripartite motif (TRIM) proteins are the largest subfamily of Ring E3-ligases. The members of TRIM family proteins are involved in a wide range of cellular processes, including signal transduction [1], transcription [2], cell cycle [3], apoptosis [4], and develop-ment [5]. A large proportion of TRIM proteins in mammals participate in innate immune responses, which regulate the transcription of nuclear factor Kappa-B and interferon regulatory factors [6]. TRIM proteins are crucial regulators in humans and their dysfunction is associated with cardiovascular [7], neurological [8], immunological, musculoskeletal, developmental disorders [9] and various types of cancer [10,11]. TRIM proteins have been well studied in mammals but the available knowledge for fish species is very limited. Recent studies have shown that several TRIM proteins of teleost species are also significantly induced during antiviral infections and play a key role in innate immune response [12,13]. A study on Zebrafish revealed that the TRIM protein is also involved in the developmental process [14].

The first TRIM protein was described as *Xenopus laevis* nuclear factor 7 in African frog [15]. Later, several studies attempted to identify and characterize these proteins in different species. The TRIM proteins are characterized by the presence of highly conserved three N-terminal region domains; one RING domain, one or two zinc-finger domains called B-boxes (B1 box and B2 box), and a coiled-coil domain collectively called “RBCC” [16]. The RING domain is a zinc-binding domain containing cysteine and histidine-rich residues that are combined in a unique cross-brace manner forming a *ββα* fold [17]. It catalyzes E3 ligase activity by binding to ubiquitin-loaded E2 enzyme and promoting the transfer of ubiquitin to a target protein [18]. The size of the RING core is around 40 to 60 residues [6,19]. The B-boxes domains are structurally similar to the RING domain but contain distinct cysteine and histidine residues [20]. Some TRIM proteins have only a single B2 box domain, while others have both B-boxes domains. The coiled-coil domain is the last characterized domain containing 100 residues [21]. The coiled-coil domain is responsible for forming dimers and higher-order oligomers of proteins. The higher-order oligomerization is important in the formation of stable complexes with other proteins, which are important for their function [22]. TRIM proteins also have different C-terminal domains responsible for the structural variations [21]. Interactions between members of the TRIM family and other proteins are often mediated through the Coiled-coil and C-terminal domains [23].

Common carp (*Cyprinus carpio*) is a specie from the Cyprinid family and is economi-cally very important, accounting for 14% of fresh aquaculture production in the world [24]. In the past decade, significant efforts have been made to characterize and understand the molecular mechanism involving the antiviral immune response of TRIM genes in teleost species. The unprecedented and unique expansion of the TRIM family was highlighted in fish and assigned the name finTRIM (fish novel TRIM [FTR]) [25]. The finTRIM has the same architecture containing RING, B-boxes, and coiled-coil domain in N-termini and variable-length C-terminal domains, including PRY-SPRY (B30.2), which is found in most of the TRIM with an antiviral function [26]. Some TRIMs from common carp and zebrafish were also studied to analyze the detailed mechanisms of these genes in response to virus infection [13,27]. These include TRIM47 homolog from common carp that restricted Spring Viremia of Crap Virus (SVCV) by decreasing mRNA level of SVCV gene and increasing type 1 interferon [27]. Similarly, overexpression of common carp TRIM32, FTR36, and zbTRIM25 from zebrafish positively regulates the innate immune response [13,28], leading to interferon production.

In humans and other tetrapods, the TRIM family of proteins has been extensively studied, however, the knowledge of TRIMs in teleost species, particularly the common carp is still very limited. A genome-wide investigation of TRIM family members in common carp was therefore conducted. The pairwise distance among teleost TRIM proteins, phylogenetic relationships, exon-intron arrangements, prediction of protein domains, physicochemical properties, discovery of motifs, gene localization, and protein secondary and tertiary structures were predicted. In addition, Gene Ontology, KEGG, and Reactome pathway analysis were also performed to understand common carp TRIM gene functions and pathway regulation. This study will help to further understand the evolutionary aspects, sequence similarity, characterization, and role of the TRIM family in common carp.

## 2. Methodology

### 2.1. Retrieval of TRIM Genes and Protein Sequences

According to our understanding, the NCBI database does not contain an appropriate nomenclature or gene annotation for the common carp TRIM family. Some TRIM family members were labeled as “finTRIM” and “FTR”, while others were mentioned as “E3 ubiquitin-protein ligases like TRIMs” and “tripartite motif-containing”. All sequences were retrieved by using these terms. The TRIM family members were renamed common carp TRIM abbreviated “CcTrim”. A total of 44 TRIM family members were found well annotated. The genomic sequence, coding sequences, and protein sequences of these TRIMs were retrieved from NCBI (https://www.ncbi.nlm.nih.gov/, accessed on 15 January 2022) and Ensemble (https://asia.ensembl.org/index.html, accessed on 15 January 2022). Interestingly, most of these TRIM members were also found curated in other teleost species. So, the sequences of similar TRIM members from zebrafish, rainbow trout, pufferfish, channel catfish, fugu, and swamp eel were also retrieved. The sequences of TRIM proteins from the genome of Human, Mouse, Cow, Dog, Cat, and Chicken were also downloaded for global phylogeny analysis. Most TRIM genes code for at least two isoforms whereby, the sequence of larger isoforms was retrieved for further analysis.

### 2.2. Sequence Alignments, Pairwise Distance, and Phylogenetic Analysis

MEGAX was used to perform multiple sequence alignment (MSA) of TRIM proteins by using the MUSCLE algorithm with default parameters (https://www.ebi.ac.uk/Tools/msa/muscle/, accessed on 15 January 2022) [29]. After alignment, the phylogenetic trees were constructed using the neighbor-joining (NJ) method based on pairwise deletion, the Poisson substitution model, and 1000 bootstrap replicates implemented in the MEGAX software [30]. The global phylogenetic tree was built using the TRIM proteins of the common carp, zebrafish, channel catfish, fugu, pufferfish, rainbow trout, swamp eel and tetrapods (human, mouse, cow, dog, and chicken). Phylogenetic tree of CcTrim proteins was also built to determine the evolutionary relationship with each other. A pairwise distance was calculated to further evaluate and deduce the evolutionary divergence of the TRIM family members of teleost species, including common carp, zebrafish, channel catfish, fugu, rainbow trout, pufferfish, and swamp eel. The heatmap of distance matrix and cladogram for TRIMs of teleost species was generated by using R package pheatmap (http://cran.nexr.com/web/packages/pheatmap/index.html, accessed on 15 January 2022) [31].

### 2.3. Gene Structure and Conserved Motif Discovery of CcTrim Proteins

The CcTrim proteins were subjected to the Multiple Expectation Maximization for Motif Elicitation (MEME) web server (http://meme-suite.org/tools/meme, accessed on 15 January 2022) [32]. The MEME online tool was used for the identification of conserved motifs by using the following parameters: (1) number of repetitions set to unlimited, (2) maximum number of motifs set to twenty, and (3) the ideal motif length was selected between 6–100 residues. The similar sequence motifs mediate a common function in a diverse TRIM protein family. Gene Structure Display Server (http://gsds.gao-lab.org/, accessed on 15 January 2022) [33] was used for the analysis of introns and exons composition of the CcTrim family genes.

### 2.4. Protein Domains Prediction and Generation of Sequence Logo

The domain information of TRIM proteins in common carp was obtained through SMART (http://smart.embl-heidelberg.de/, accessed on 15 January 2022) [34] and Pfam database (https://pfam.xfam.org, accessed on 15 February 2022) [35]. The prediction of protein domain is very important for the identification of a particular function and interaction with other proteins. The N-terminal region contains conserved domains that fulfill the rule of TRIM proteins. A web based application called WebLogo (https://weblogo.berkeley.edu/, accessed on 15 January 2022) [36] was used to generate the sequence logo of the two most conserved domains of teleost TRIM proteins. Each sequence logo was generated by using representative sequences of RING and B-box domains.

### 2.5. Physico-Chemical Properties and Subcellular Localization

The Physico-chemical properties of the CcTrim proteins were computed using the ExPASy ProtParam tool (https://web.expasy.org/protparam/, accessed on 15 January 2022) [37]. The subcellular localization of the selected CcTrim proteins was predicted by using the Balanced Subcellular Localization Predictor tool (BaCelLo) (http://gpcr.biocomp.unibo.it/bacello/pred.htm, accessed on 15 January 2022) [38] and CELLO online server v.2.5 (http://cello.life.nctu.edu.tw/, accessed on 15 January 2022). The protein subcellular localization is pivotal to understanding the mechanism of protein function.

### 2.6. Secondary and Tertiary Structure Prediction

The secondary structure predictions of the CcTrim proteins were performed by using PSIPRED 4.0 (http://bioinf.cs.ucl.ac.uk/psipred/, accessed on 15 January 2022) [39]. Protein secondary structure prediction provided the proportion of structural elements of α helices, β sheets, and coil-coil elements. The tertiary structures of CcTrim proteins were modeled by using an improved deep learning based modeling method RoseTTAFold available at the Robetta protein structure prediction service (https://robetta.bakerlab.org/, accessed on 15 January 2022) [40]. The structures were visualized and superimposed by using PyMol (v2.5) (https://pymol.org/2/, accessed on 15 January 2022). The predicted structures for the CcTrim proteins were assessed for quality by using the Structure Analysis and Verification Server (SAVES) v6.0 (https://saves.mbi.ucla.edu/, accessed on 15 January 2022). This server (v6.0) provides numerous tools for the quality assessment of proteins, including PROCHECK, ERRAT and verify 3D. The Qmean4 value was also identified using a Tool based on the Swiss model (https://swissmodel.expasy.org/qmean/, accessed on 15 January 2022).

### 2.7. Gene Location and Protein-Protein Interactome Analysis

The precise location of CcTrim genes helps in determining the distance from other genes and locating whether the gene is present on the 5 prime end or the 3 prime end of the chromosome. Phenogram, an online tool developed by Ritchie Lab (http://visualization.ritchielab.org/phenograms/plot, accessed on 15 January 2022) [41] was used to visualize the exact location of CcTrim genes in a particular common carp chromosome. An association network of CcTrim proteins was established by using the STRING database version 11.5 (https://string-db.org/, accessed on 28 February 2022) [42] with a default value of confidence. The maximum number of interactions were set to not more than 30 and *Danio rerio* (zebrafish) was used as a query organism. Further, Cytoscape (https://cytoscape.org/, accessed on 15 January 2022) (V3.90) [43] was used for better visualization and annotation of the network to gain an enhanced understanding.

### 2.8. Gene Ontology, KEGG, and Reactome Enrichment Analysis

Blast2Go module implemented in Omicsbox 2.0.36 (https://www.biobam.com/omicsbox/, accessed on 15 January 2022) was used to generate Gene Ontology (GO) mapping from GO database. The protein sequence of CcTrim in fasta form at was used to run blast2go that performed high- throughput blast and interproscan. The BLAST and domain hits were imported into OmicsBox, which gives GO terms in the form of a table. Reactome is curated and peer-reviewed pathway database available for most model organisms. Common carp specie is not curated yet thus functional enrichment from String Network was used to explore the role of CcTrim proteins in Reactome biological pathways. For Kyoto Encyclopedia of Genes and Genomes pathway (KEGG pathway) analysis “KAAS-KEGG automatic annotation server” (https://www.genome.jp/kegg/kaas/, accessed on 15 January 2022) was used to generate KO (KEGG Orthology) assignments that characterize individual gene functions and reconstruct KEGG pathways.

## 3. Results

### 3.1. Global Phylogenetic Analysis of TRIM Proteins

**Figure 1:**
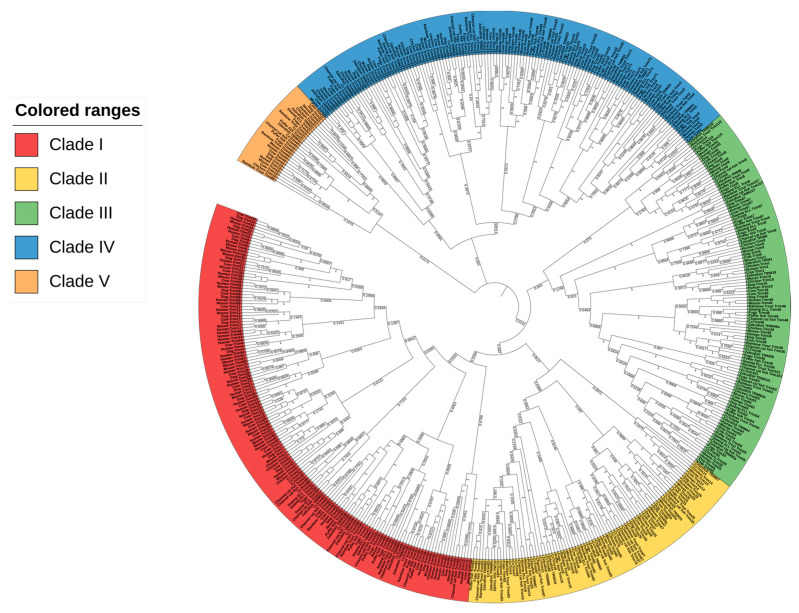
Global phylogenetic analysis of the full-length sequence of TRIM proteins from teleost species (common carp, zebrafish, fugu, pufferfish, channel catfish, rainbow trout and swamp eel) and tetrapods (Human, Mouse, Cow, Cat, Dog, and Chicken). MEGAX was used to construct a phylogenetic tree by using neighbor-joining (NJ) method and a bootstrap value of 1000 replicates. The phylogenetic tree is divided into five different groups based on clades of TRIM proteins. Each group was marked with unique color.

A global phylogenetic tree was constructed to elucidate the evolutionary relationship of CcTrims with the TRIMs of human, mouse, cow, dog, chicken, and multiple teleost species such as common carp, zebrafish, fugu, swamp eel, channel catfish, rainbow trout, and pufferfish. Five different groups were identified in order to explain the phylogenetic tree and compare the TRIMs of teleost and tetrapods. The global tree is represented in Figure 1, where the labels of each group are highlighted using a different color. The resulting global phylogenetic tree (Figure 1) depicts that different TRIMs from the same species did not cluster together but rather appeared to be dispersed within different clades. This is due to the fact that TRIMs showing similarities in sequence and structures cluster together thus, occupying adjacent branches of the tree.

Overall, the global phylogenetic tree splits the TRIM proteins into five different clades with numerous sub-clades. It is notable that, there are numerous sub-clades in every group connected to a common node. Clade 1 contains TRIMs from all selected species and is comprised of 153 leaves highlighted with red color in a tree. A large proportion of TRIMs from this clade contains RBCC-B30.2 domains which were clustered together. In this group, a total of nine CcTrims are present. Hence, the group is dominated by tetrapod TRIMs containing RBCC-B30.2 domains such as TRIM21, 34, 40, and 7 thus, indicating that these members are closely related to each other. Conversely, some common carp TRIMs such as TRIM62 and 35 were clustered to both teleost and tetrapod TRIM members. Moreover, the TRIMs 108 and 109 of teleost, which are not recognized in tetrapods were found adjacent to TRIMs 62 and 69 of both mammals and teleost species. Additionally, the Clade II highlighted yellow in Figure 1, contains a total of 85 leaves. Among these sixteen leaves are CcTrims. The TRIMs of this group also contain RING, B-box, coiled-coil, PRY, and SPRY domains that are present in both teleost and higher vertebrate members. All the members of this group lacked other diverse C-terminal domains. TRIM47 from human, mouse, cow, and dog were placed adjacent to each other. Some TRIMs including TRIM14 and 16 lacked the RING domain but display sequence identity with clade II members such as TRIM25, 65, 86, and 87 as shown in Figure 1. Furthermore, common carp TRIM25 and 65 showed maximum identity with their respective homologs both from teleost and tetrapod TRIM members. These results show some members of TRIMs are conserved in both teleost and higher vertebrate species. The detailed results of each TRIM can be further seen in Figure 1. The phylogenetic analysis showed that the evolution among similar TRIM (homologous) members is not very divergent and mostly originated from a common node. Overall, the TRIM members of clade I and II are composed of RBCC and this domain set is conserved in N-terminal with C-terminal PRY and/or SPRY only. The clustering of these members into two groups provides evidence of maximum homology in sequences among the TRIMs that are comprised of RBCC-B30.2 domains in teleost and higher vertebrate species.

Clade III contains a total of 114 leaves and among these nine are CcTrims which is more diverse (highlighted green in Figure 1). Unlike clade I and clade II, most TRIM proteins present in this group are recognized and identified with a combination of specific C-terminal domains such as fibronectin type 3 (FN3) and trans-membrane (TM) domains. The TRIM members such as TRIM1, 9, 18, 36, 46, and 67 contain RBCC with additional B-box, BBC, and FN3 domains. Moreover, another distinct C-terminal trans-membrane domain was observed in TRIM101 of teleost species. In addition, TRIM54 and 55 of this group are comprised of RING, B-box, and coiled-coil only and lack C-terminal domains. Similarly, TRIM8 and 52 were also found adjacent, which shows non-conserved RBCC domains in their N-terminal and the absence of C-terminal region domain. Common carp TRIM, including CcTrim9, CcTrim36, and CcTrim46 specifically contain FN3 domain and cluster with their respective TRIM homologs of both teleost species and tetrapods. This indicates the clade III members are conserved in regards to occurrence of functional domains between teleost and tetrapods. Most members of this group are not comprised of C-terminal domains and the domain’s architecture distinguishes these members from other TRIM members. Clade IV also contains TRIM protein from both teleost and tetrapods. It contains a total of 133 leaves, and among these eight are CcTrims. The TRIM proteins of this group are found in all selected species. The diversity of C-terminal domains is maximum in these members, as compared to other group members. The TRIM2, 3, and 45 contain FLMN domain, while other members such as TRIM23, 33, and 66 from teleost and tetrapods consist ARF and PHD-BROMO domains respectively. Moreover, TRIM59 of higher vertebrate species show only RING on N-terminal and transmembrane domain on C-terminal thus, completely lacking other domains. This clade also contains TRIM20 homologs, which contain a PYRIN instead of RING domain. Thus we can speculate that the functional domains of this clade members are different but the distance or mismatch at aligned positions between the sequences may be very less. Overall, the members of these groups showed a diverse domain arrangement at both ends and share a common phylogenetic origin.

Clade V has a small cluster with a total of 27 leaves which were dispersed into several sub-clades. Some members of this group have a conserved B-box, coiled-coil domain in N-terminal region, while MATH and PML domains in C-terminal region as observed in TRIM37 and 19. Interestingly, some members including TRIM44 of this group contain only RING with either B-box or coiled-coil only and lack C-terminal domains. The unique combination in N-terminal region and distinct domain found in C-terminal disclose that the sequence identity is also different from other TRIMs. The cross-species comparison revealed the resemblance and evolutionary divergence of CcTrims with other teleost and specialized organisms. Most of the TRIM members clustered with tetrapods have a variety of C-terminal domains, which are conserved and recognized in both teleost and tetrapods. Conversely, the TRIMs of teleost or vertebrates that cluster among each other contain RBCC with SPRY and PRY domains in different combinations. Most members of clades I and II have RBCC-B30.2 domain arrangements, while clades III, IV, and V contain discrete C-terminal domains.

### 3.2. Teleost TRIM Proteins Are Not Evolutionary Distant

A heatmap was generated from pairwise distance values to elucidate the resemblance of TRIM proteins between common carp and other teleost species. A total of 221 TRIM protein sequences of common carp, zebrafish, fugu, pufferfish, rainbow trout, channel catfish, and swamp eel were used in this analysis, which gives a comparison of all-to-all sequences.

**Figure 2:**
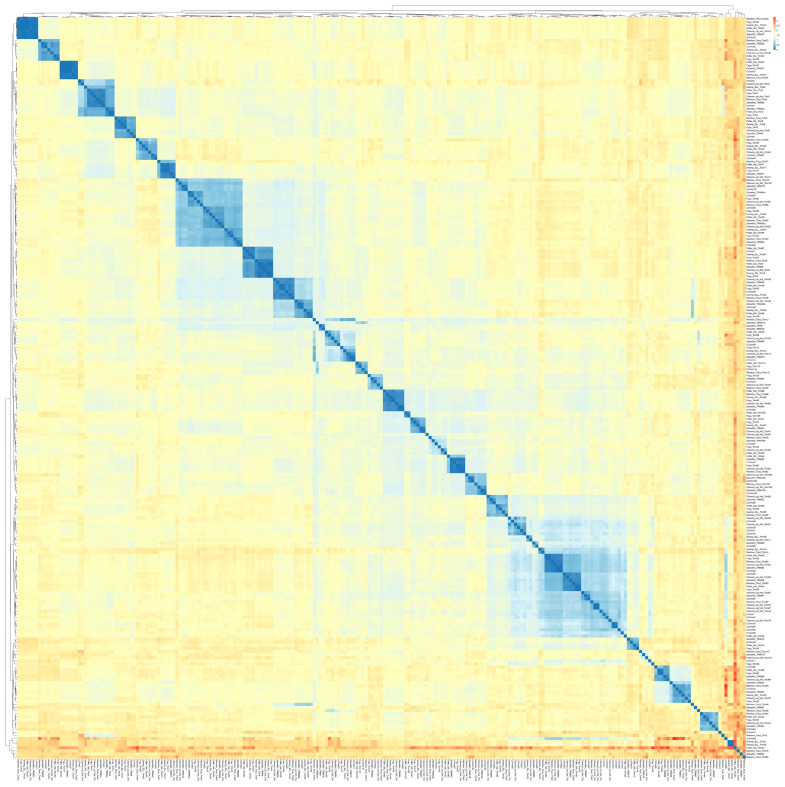
Heatmap based on the pairwise distance among amino acids of TRIM proteins from different teleost species (common carp, zebrafish, channel catfish, pufferfish, rainbow trout, fugu, and swamp eel). Different colors illustrate the heatmap ranging from blue (less distance value) to red (highest distance value). Each protein sequence was compared to find the mismatch at align position between common carp TRIMs and other teleost TRIMs. This analysis allowed the comparison of all sequences vs. all sequences. The cladogram across the top and left of the heatmap shows the evolutionary divergence based on distance values. Names are listed on the right and bottom of the diagram.

A different color scheme is illustrated in the heatmap (Figure 2), where blue squares indicate less distance value (or exactly similar) and red squares represent high distance value. The result showed that most TRIM proteins from teleost species were not evolutionary distant and the fraction of mismatch at aligned positions was small. The heatmap in Figure 2 represents a lower proportion of red squares, which indicates a lesser number of highly distant sequences. Some red spots were observed, but pufferfish TRIM33 were observed highly distant from majority of teleost TRIMs. Furthermore, the clustering of rainbow trout TRIM66, zebrafish TRIM97, rainbow trout TRIM16, swamp eel TRIM21, 25 and CcTrim99 generate some prominent red squares. The cladogram of these sequences was also constructed that places closely related sequences under the same node. Several clusters of cladogram were observed that revealed the evolutionary divergence of teleost TRIM proteins. TRIMs including TRIM82, 83, and 87 from all teleost species, TRIM16 and 37 from channel catfish, and CcTrims29, 56, 79, and 97 clustered together in the cladogram a as shown in blue in the lower right part of the heatmap. Similar to other teleost species, TRIMs from common carp were also distributed in many clusters, thus, indicating that the CcTrims are evolutionary divergent. Furthermore, Some TRIM members, including TRIM9, TRIM36, TRIM46, TRIM54, TRIM55, and TRIM63 also clustered together. These TRIMs are evolutionary conserved, which means they are similar in sequence and domain architecture which are recognized in most vertebrates. Additionally, the domains of these TRIMs in the C-terminal region are specific to these members, for example, FN3 in TRIM9, 36, and 46. The clustering of these TRIMs in that region also generates another less visible blue region on the top left part of a heatmap. Most teleost TRIMs having designated a specific ortholog number are present on adjacent branches of clusters and correlate with each other based on sequence similarity. Overall the light yellow squares present in the heatmap shows the TRIMs across teleost species are closely similar and not very distant.

### 3.3. Phylogeny, Gene Structure, Motifs, and Domain Architecture of CcTrim

**Figure 3:**
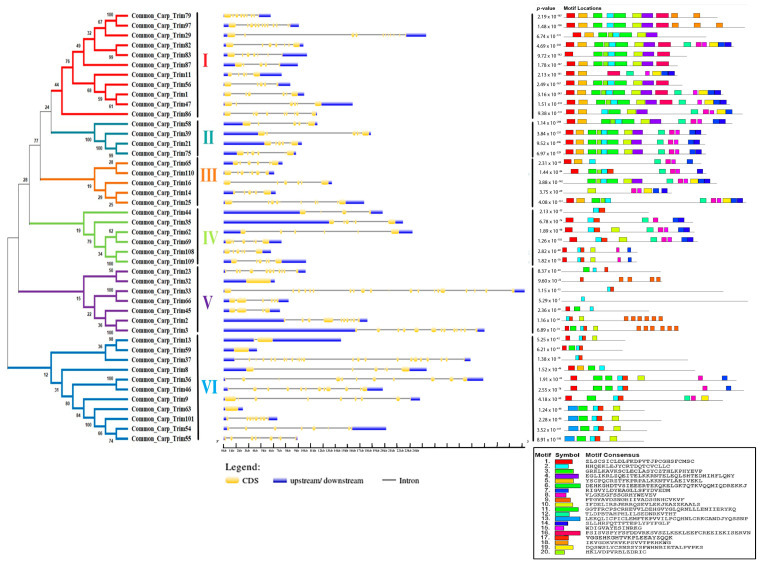
Phylogenetic relationship, gene structure, and motif composition of TRIM family in common carp. The phylogenetic tree of CcTrim proteins were classified into six groups based on clusters generated through phylogenetic analysis. Each cluster is marked with different color and designated as cluster 1–6. Next the blue color represents 5 and 3 prime end untranslated region, the black lines show introns and the yellow color represents exons of genes. Moreover, motif composition of the CcTrim proteins elucidated twenty different motifs that are presented in a color box having specific amino acid pattern. The black line represents non-conserved sequences. At the bottom, the unique color symbol of motifs 1 to 20 with consensus sequence is shown.

**Figure 4:**
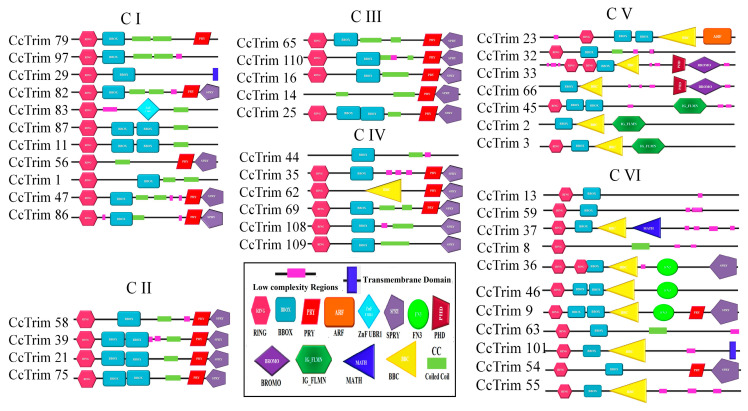
Complete Structure of CcTrim proteins from N-terminal to C-terminal region. Each domain was symbolized and represented with unique geometric shapes and colors.

A phylogenetic tree was constructed to further elucidate the evolutionary relationship of CcTrim proteins with each other. The proteins were distributed in different clades, thus, generating six clusters shown in Figure 3. Based on phylogenetic clusters, the CcTrim proteins are classified into six groups designated as group 1–6. Clusters 1 and 6 are the largest and contain an equal number (11) of CcTrim proteins. The clusters 2 and 3 are smaller, with four and five CcTrim proteins. Whereas, clusters 4 and 5 contain six and seven CcTrim proteins respectively. The members of each cluster correlate in terms of the domain architecture. However, the domains identified in different CcTrim clusters showed a different combination of RBCC and are not precisely similar to TRIMs of tetrapods. In this study, computational approaches indicate that the B-box 1 domain is slightly larger, ranging from 46 to 60 residues, whereas the B-box 2 domain is comprised of approximately 40 residues. Moreover, the alignment shows the sequences of both B-box domains are not conserved with each other. The existence of B-box domains in TRIM proteins follows the same pattern as present in mammals. The B-box 1 in CcTrims always precedes the B-box 2. Thus, it can be speculated that B-box 1 and B-box 2 of CcTrims are not identical copies of one another. The detailed information on different domains present in each CcTrim protein is shown in Figure 4. TRIMs of group 1 contain RING, B-box, coiled-coil, PRY, SPRY, TM and ZNF-UBR1 domains. The C-terminal region of this group is not very diverse, only CcTrim29 and CcTrim83 specifically display transmembrane (TM) and Ubiquitin Protein Ligase E3 Component N-Recognin 1 (UBR1) type zinc finger (ZNF-UBR1) domains respectively. The ZNF-UBR1 domain targets the N-end rule pathway for substrate degradation [44] and may not be reported in the mammalian TRIM proteins. The most frequently found domains after RING, B-box, and coiled-coil are PRY-SPRY, that are also known as B30.2. The PRY and SPRY domains either exist individually or side-by-side and are involved in protein-protein interactions, retroviral restriction, innate immune and cytokine signaling [45]. The C-terminal TM domain was found in CcTrim29, which might be localized in the endoplasmic reticulum to restrain inflammatory response and other membrane trafficking roles [46].

All members of group 2 and group 3 display RING, B-box, coiled-coil, PRY, and SPRY domains except CcTrim14 and 16, which did not have RING domain. Most TRIM proteins with B30.2 domain have been reported to play a role in antiviral response in mammals [45]. Similar to group 2 and group 3, TRIMs of cluster 4 also contain RING, B-box, coiled-coil, PRY, and SPRY domains and lack C-terminal diverse domain. However, the domain combination is different in other members of cluster 4 such as CcTrim44 does not have a RING domain. In addition, the CcTrim62 is the only member of this cluster that contains a B-box C-terminal coiled-coil region (BBC). BBC domain is associated with ring finger proteins and GTP-binding proteins. Domains of group 5 members are more diverse both in N- and C-termini. The C-terminal region of CcTrim45, 2 and 3 display filamin-type immunoglobulin (IG-FLMN), while CcTrim2 and CcTrim66 are lacking RING domain. IG-FLMN are rod-like structures in the actin-binding cytoskeleton protein called filamin and are assumed to play role in the embryogenesis and developmental process of zebrafish [47]. Two TRIMs (CcTrim33 and 66) contain a pair of plant homeodomains (PHD) associated with BROMO domain. Moreover, the CcTrim23 is unique among all TRIMs as it is the only member that contains ADP-ribosylation factor (ARF) domain. ARF domain performs GTPase activity and facilitated intracellular trafficking while the PHD and BROMO are essential for transcriptional repression and DNA damage response [48]. The majority of members of group 6 contain RING and B-box, while the occurrence of coiled-coil domain is less frequent. Two members CcTrim13 and 59 contain RING and B-box only. Several other members of this group are comprised of distinct domains, including the Meprin and TRAF Homology (MATH) domain in CcTrim37 and Fibronectin type 3 (FN3) in CcTrim9, 36 and 46. The FN3 domain contains binding sites for DNA and heparin, while for the C-terminal subgroup one signature (Cos) domain was not predicted by SMART. In mammals, the domain arrangement of TRIM9, 36, 46 are RBCC on N-terminal and COS-FN3-B30.2 in C-terminal region of proteins. In comparison, the arrangement showed by CcTrim9, 36 and 46 are RBCC-FN3-B30.2 lacking the COS domain. MATH domain was only found in CcTrim37, that plays an important role in ubiquitination, proteolysis, and protein processing [49]. A unique pattern is also observed in the N-termini of CcTrim33 and CcTrim36, consisting of two RING domains. Some TRIMs from teleost have been identified to play role in innate immunity but in-vitro studies are still required on several TRIMs to identify their exact functions.

Exon and intron structural diversity often plays an important role in the evolution of the gene family. Analyzing intron and exon structures helped to gain insights into CcTrim genes. The size of a gene mainly depends on the size and number of introns. Among the CcTrim family, CcTrim33 (24.257 kb) is the largest gene, while the smallest gene identified was CcTrim63 (1.551 kb). The number of introns in CcTrim genes CcTrim56 to 23 and CcTrim37 ranged from four. The gene structure of the same members is not precisely similar; however, the number of introns and UTRs in some adjacent tree members are correlated. For example, the number of introns was four in members of cluster 1, including CcTrim11, CcTrim56, and CcTrim87. Similarly, all members of cluster 2 contain five introns, and the members of cluster 4 showed five introns except CcTrim44, which possesses three introns. Variation occurred in the gene structures of different group members, these substantial differences and structural diversity is caused by the number and size of UTRs, exons, and introns of the gene. UTRs lengths of cluster 4 members, including CcTrim44 (≈9 kbp), CcTrim35 (≈12.5 kbp), and cluster 5 members such as CcTrim3 (≈16.5 kbp) and CcTrim2 (≈8 kbp) are longer as compared to other group members as demonstrated in Figure 3. All the genes exhibit 5’ end or 3’ end UTRs in their peaks indicating a complete structure of the gene.

During the investigation of conserved sequence patterns, a maximum of 20 motifs were discovered that correspond to some domains. The number of residues in motifs of CcTrims ranged from 14 to 41 amino acids. Motif1 presented in red boxes corresponds to RING domain with amino acid pattern “ZLSCSICLDLFKDPVTJPCGHSFCMSC”. Motif2 with amino acid pattern “HHQEKLEJYCRTDQTCVCLLC” is represented in cyan color box in Figure 3 which corresponds to B-box domains. The consensus pattern of motif3 is also present in B-box domains. Moreover, coiled-coil domain contains the sequence pattern of motif4 and motif10. The consensus protein pattern of motif8 is present in both PRY-SPRY, while motif19 is only present in SPRY of some TRIMs. Two motif patterns are predicted in BBC domain such as motif17 and motif6. The consensus sequence pattern of motif6 is one of the longest consisting of 40 residues and is present at the end of B-box and start of coiled-coil. The domains that correspond to each motif are presented in Table 1 and the pattern is given in Supplemental Materials S1, Table 2. Motif1, 3, 10 and 17 are present in more than half of CcTrim members. Moreover, some of the motifs are repeated many times in a single protein sequence, e.g., motif9 is repeated six times in CcTrim2, CcTrim3, and four times in CcTrim32. Motif18 also occurs twice and thrice in CcTrim79 and CcTrim97, respectively. Overall, members of clusters 1, 2, and 3 are comprised of close and comparable motifs relative to the bottom three groups. The function of these predicted motifs is still unknown, and detailed studies on TRIM protein interactions may provide some clues to the function. 

### 3.4. Physico-Chemical Properties and Subcellular Localization

The physico-chemical properties of proteins such as amino acid size, molecular weight, instability index, aliphatic index, and Grand Average of Hydropathy (GRAVY) were predicted using an online tool that gives an insight into protein behavior. The detailed values of physico-chemical properties of CcTrim proteins are provided in Table 2. The number of amino acids in CcTrim proteins ranges from 319 to 1229. Most of the sequences contain 400 to 700 amino acids. Approximately, 75% of the CcTrim proteins have molecular weights within the range of 45–75 kDa. These results suggest most proteins are in the best range to perform molecular analysis such as making expression vectors and immunological characterization. The instability index reveals that only four (CcTrim2, CcTrim9, CcTrim82, and CcTrim97) proteins are stable and the rest are unstable. The stable protein include two members from cluster 1 (CcTrim82 and CcTrim97), one member from cluster 5 (CcTrim2), and one member from cluster 4 (CcTrim9). Majority of the stable proteins are in the range of 600 to 700 amino acids and among these the CcTrim82 are localised in nucleus. Isoforms of promyelocytic leukemia (PML)/TRIM19 proteins also range from 48–97 KDa conferring apoptotic and antiviral activities [50]. Theoretical isoelectric points (PI) ranges from 4.63 to 8.86, where 14 of the proteins (32.0%) were explored to be basic (*PI* > 7) and 29 (62.0%) were observed to be acidic (*PI* < 7). The value of GRAVY for all proteins is negative, revealing hydrophilic nature. Aliphatic amino acid indices revealed differences in thermal stability ranging from 69.48 to 91.39. The subcellular localization of the CcTrim protein was predicted to be localized in the cytoplasm and nucleus, indicating a diverse cellular role. Previous studies on TRIM19 of mammals form nuclear aggregates and is termed as PML nuclear bodies (PML-NBs) that indicate the function of TRIM inside the nucleus [51]. TRIM19 is an essential component of NBs, which are sites of transcription regulation and for localization of several viral components. Furthermore, TRIM19 negatively regulates IFN*γ* signaling by inhibiting STAT1, and the cytoplasmic isoform of TRIM19 also works as an adaptor to bind to TGF*β* receptor [52]. Some members of cluster 5 and 6 were also predicted to be localized in the nucleus, which may perform similar functions already described in mammals. Considering existing literature of TRIMs from mammals and physico-chemical properties listed in the Table 2, we can easily speculate these proteins might also performed diverse cellular function.

### 3.5. Sequence Logo of RING and B-Box Domains in Teleost TRIMs

**Figure 5:**
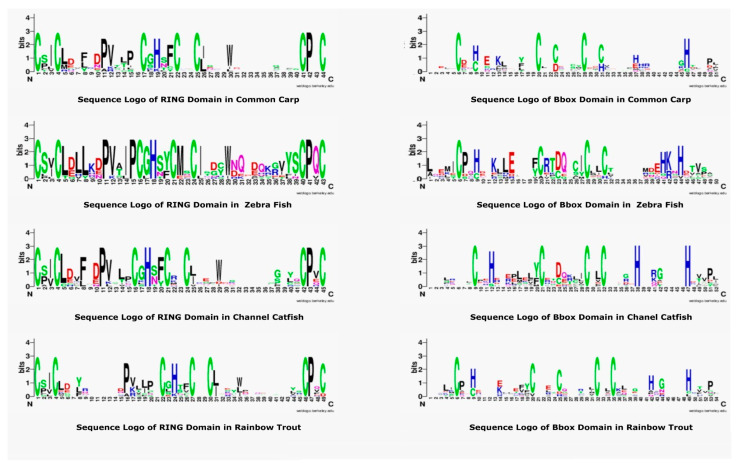
Sequence Logo representation of common carp, zebrafish, channel catfish, and rainbow trout of RING and B-box domain sequences from TRIM proteins. In each logo, the *X*-axis indicates the conserved amino acids displayed with one letter and specific color. The height indicates the relative frequency of amino acids at that position. The sequence logos of these domains were constructed by using the Weblogo platform.

The RING and B-box are most conserved and important domains of TRIM proteins. A sequence logo of these domains was generated and then compared with other teleost species to explore the conserved amino acids. The most conserved residues are cysteine and histidine, as shown in Figure 5, that are usually attached to zinc ions to stabilize the tertiary conformation. The pattern of seven cysteine residues and one histidine residue is present in the RING domain of all teleost TRIMs. Some residues, including proline, were also conserved, that may be responsible for making motifs specific to this region. Comparative analysis of the B-box sequence logo revealed cysteine and histidine were conserved, but a slight variation exists. The RING sequence logo revealed the relative frequency and number of histidine are seven. However, B-box sequences from common carp and rainbow trout showed five conserved cysteines, while zebrafish and channel catfish showed four conserved cysteine residues. The position of amino acids was also different, which may be responsible for performing different functions and forming the tertiary structure conformations.

### 3.6. Secondary and Tertiary Structure Prediction of CcTrim Proteins

In terms of abundance, the CcTrim proteins were predominantly composed of coiled-coil regions followed by α helices. The occurrence of β-sheet elements was significantly less. The members of cluster 1 have maximum similarity on coiled-coil content ranging from 44–55%, however, the difference was observed in predicted α helices and β sheets ranging from 29–53% and 3–20%, respectively. The percentage of predicted secondary structure is quite similar among the members of clusters 2, 3, and 4. The minimum and maximum ranges for these clusters of α helices, β sheets, and coiled-coil was 33–43%, 10–19%, and 40–53%, respectively. The detail of cluster-wise secondary structural elements of each CcTrim is given in the Supplemental Materials S1, Table S4. A substantial difference in the values has been observed in the percentage of secondary contents among the members of cluster 5 and 6. The lowest value of β sheets was predicted to be 4% for both CcTrim33 and 59. Likewise, some members of cluster V and VI revealed highest range of coiled-coil segments such as CcTrim66 showed 76% and CcTrim62 manifested 62%. The range of α helices in clusters 5 and 6 are comparable to other members of cluster 1–4.

**Figure 6:**
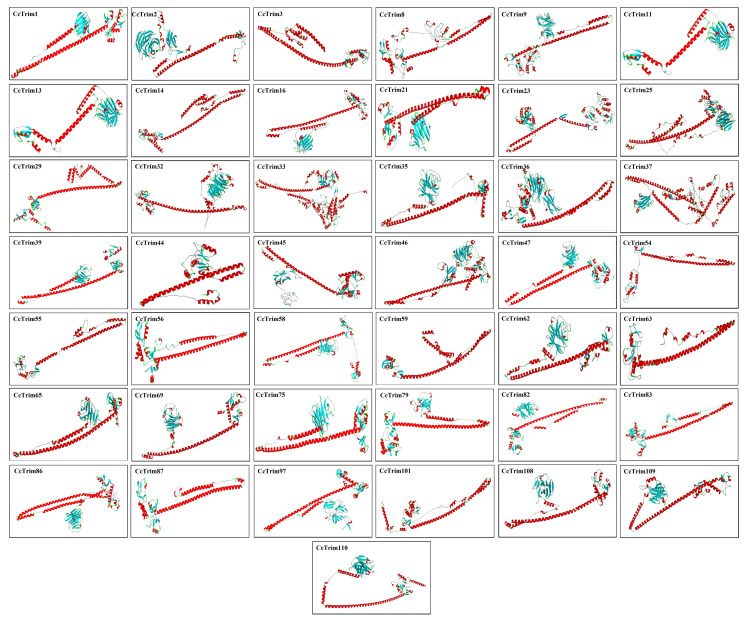
Predicted 3-D Superimpose structure of common carp TRIM proteins. The Red colour in the 3D structure indicates α helices, cyan colour represents the β sheets, white colour indicates the loop regions whereas, green colour indicates turns.

The known structure of a sequence can serve as a template to model the structure of the target protein by using different computational approaches as mentioned in methodology. The built structures of CcTrims are presented in Figure 6 and (Supplemental Materials S1). The tertiary structure of most CcTrim proteins showed antiparallel coiled-coil segments separated by helical conformation at both ends. The reliability of all predicted protein models was evaluated through Qmean, ERRAT, and Verify3D. The Qmean scores ranged from −3.46 to 2.45 and ERRAT percentage of model proteins was observed from the lowest 78.2% to the highest 100%. The verify 3D percentage was observed between 28.94% to 73.16%. The high score of Qmean and ERRAT indicate good quality of the models. Ramachandran plot for all proteins display the ranges of most favored regions from 77% to 93.3%, whereby the additional allowed regions showed 5.6% to 18.7%, generously allowed regions 0.0% to 2.0% and disallowed regions showed 0.0% to 2.4% (Supplemental Materials S1, Table S3). Hence, all the predicted protein models were considered to be of good quality that can provide a preliminary basis for understanding the CcTrim protein structures and functions.

### 3.7. Distribution of CcTrim Genes on Chromosomes

**Figure 7:**
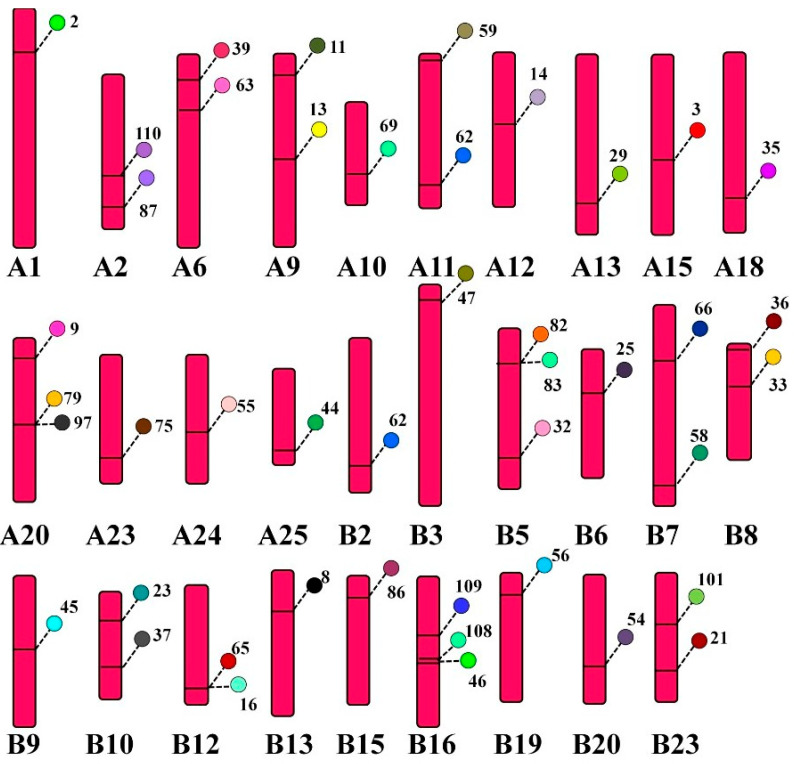
Location of CcTrim genes on 29 chromosomes loci marked with different colors. PhonoGram a web-based tool was used to visualize location and the chromosome number was indicated at the bottom of each chromosome. The gene locus is a label with particular TRIM number and is shown using a unique color.

Some of teleost species, including common carp undergo genome duplication and are included in tetraploid species. Common carp contains twenty five pairs of homologous chromosomes and each homolog is symbolized by A and B. The web-based tool PhenoGram helped to visualize the location of CcTrim genes on each chromosome. The result showed CcTrim genes were widely distributed on 29 chromosomes, and the number of genes on each chromosome varied from 1 to 3. The distribution suggests that most genes are mobile and expand independently, but some genes are linked closely. TRIMs, including CcTrim79 and CcTrim97 are linked and present in very near loci of chromosome A20. Similarly, CcTrim82, CcTrim83, CcTrim16, and CcTrim65 sit close together in chromosomes B5 and B12, respectively. These genes are likely to be translated and inherited together. Physically close genes present on the same chromosome are cosegregated together, and fewer recombination events may occur. More than half the number of individual chromosomes contain one gene, while nine chromosomes have two CcTrim genes, and three chromosomes contain three sets of genes, as depicted in Figure 7. The position of CcTrim is irregular, maximum genes were on the upper end of the chromosome arm, and some were placed in the lower arm. The genes located on the middle part of a chromosome are very few. There are twenty CcTrim genes distributed on chromosomes of homolog A and twenty-four CcTrim genes on the chromosome of homolog B. The distribution list is available in Supplementary Tables. Each gene locus is shown with a unique color.

### 3.8. Protein-Protein Interactome Analysis

Protein-protein interaction (PPI) network analysis is based on homology analysis for predicting protein interactions and function. In this study, the PPIs of CcTrim proteins were also elucidated. PPI networks are largely incomplete for non-model organisms; thus, zebrafish was selected as the preferred specie in the STRING database. The proteins that match with amino acid sequences of CcTrims appear and those proteins were selected, which showed higher identity from zebrafish genome. The names of CcTrim proteins, homologous STRING proteins and predicted functional partners are available in (Tables S1 and S2 of Supplemental Data Sheet 2).

**Figure 8:**
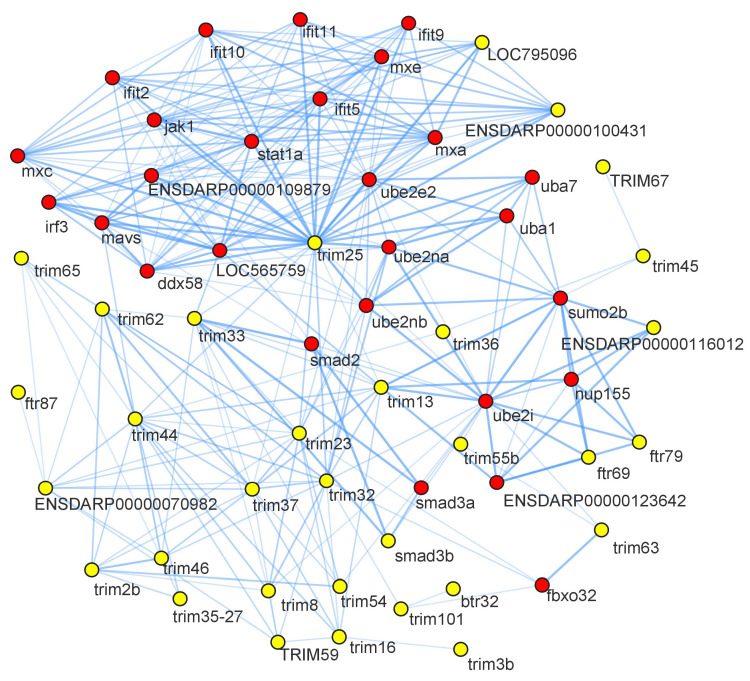
Protein-protein interaction network predictions are shown, yellow nodes indicate the CcTrim proteins and red nodes are their interacting partners. The name of node is shown as the proteins name to the string database identifier. The disconnected nodes are not present in the given figure.

The analysis generated an interaction network consisting of 73 nodes and 285 edges. Nodes represent proteins, and edges represent the interaction between the two proteins. Yellow nodes on the network indicated CcTrim proteins, and red color nodes are their interacting partners. Some of the CcTrim proteins showed higher identity with zebrafish proteins but are not curated and their interactions are not known in the STRING database. These include CcTrim1, CcTrim11, CcTrim23, CcTrim39, CcTrim47, CcTrim58, CcTrim69, CcTrim82, CcTrim83, CcTrim84, CcTrim86, CcTrim108, CcTrim109 and CcTrim110. Among these members, some of the TRIMs are only present in teleost species; thus, some experiments or curation is required to add annotation of these TRIMs in STRING database. The disconnected nodes of 14 CcTrim proteins are not shown in Figure 8. TRIM25 is well characterized, curated and experimental data is available for both mammals and teleost species. String analysis shows CcTrim25 (comprised of B30.2 domain) has a maximum number of interactions with immune signaling proteins. TRIM proteins comprised of B30.2 containing-domains mediated protein-protein interactions and are involved in innate cellular response [45]. CcTrim29, CcTrim79, and CcTrim97 interacted with common proteins such as nucleoporin (nup155), Small ubiquitin-related modifier 2 (sumo2b), and ubiquitin-conjugating enzyme 2 (ube2i). The predicted functional partners were mostly interferon-induced and regulatory proteins such as ifit2, ifit5, ifit9, ifit10, ifit11, mxa, mxc, mxe, and irf3, which mediate innate antiviral immune and inflammatory responses. Further, the interacting partners include ubiquitin system proteins (ube2na, ube2nb, ube2i), that mothers against decapentaplegic homolog, which promotes differentiation of dorsal tissue and formation of embryogenesis in Drosophila. Several TRIM proteins have been observed to interact with each other. Similarly, the interactions had been among predicted functional partners.

### 3.9. Gene Ontology

**Figure 9:**
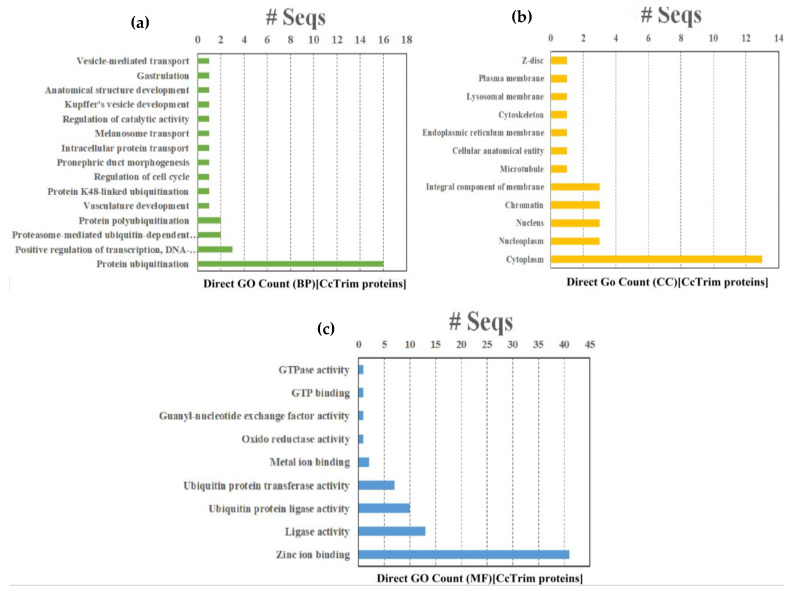
Gene Ontologies (**a**) Graph representing the Gene Ontology distribution in biological processes, (**b**) graph representing the Gene Ontology distribution in cellular components, (**c**) graph depicting the Gene Ontology distribution in molecular function.

Gene Ontology (GO), KEGG and Reactome pathway enrichment analysis are extremely helpful in determining the functional and biological relevance of a set of genes. The results of Gene Ontology (GO) analysis are divided into three different categories such as Biological process (BP), Molecular function (MF) and cellular component (CC). A total of thirty 36 GO terms were annotated from GO database. Fifteen GO terms belong to biological process, twelve are molecular function and nine belong to the cellular component category as shown in Figure 9 and Supplemental Data Sheet 2. The term (GO:0016567) protein ubiquitination was significantly enriched and annotated in sixteen CcTrim proteins in the biological process. Among MF categories the term (GO:0008270) zinc ion binding from a category of MF is highly enriched and annotated in 41 CcTrim proteins. The zinc finger motif structure that contains the zinc ion bounded with four cysteines or histidines is predominant in RING and B-box domains of TRIM family.

The CcTrims of some clusters showed similar gene ontology and are correlated. The CcTrim members of cluster 1 were involved in a variety of GO categories. Zinc ion binding is annotated in all members except CcTrim11, that showed metal binding as an alternative GO term, and six members showed ligase activity. In addition, CcTrim79 and CcTrim97 were annotated with protein ubiquitination, one of the major functions of TRIM proteins, while a term describing vascular development was also observed specific to CcTrim82 only. CcTrim members of clusters 2 and 3 were also annotated with zinc ion binding except for CcTrim14 that does not have any RING or B-box domain. This result also increases the accuracy of predicted domains and GO terms. Three member from cluster 2 also showed ubiquitination and ubiquitin protein transferase activity except for CcTrim58. Moreover, four members of cluster 3 showed ligase activity. These groups are enriched with RING, B-box, coiled-coil PRY, and SPRY domains, indicating an active role in E3-ligase activity.

The number of GO terms increases when the diversity of domains in C-terminal region increases in CcTrims. Members of clusters 4, 5, and 6 showed diverse and maximum number of annotations relative to above listed three clusters. Four members of cluster 4 were involved in ubiquitination while two members including CcTrim35 and CcTrim69 were not annotated to predict protein ubiquitination or ligase activity. Additionally, a unique function of anatomical structural development and oxidoreductase activity is shown by two cluster 4 members the CcTrim35 and CcTrim44, respectively. Three members of cluster V participate in ligase and ubiquitination activity. A number of the distinctive functions shown by CcTrim23 were intracellular transport and GTPase activity, while gastrulation, melanosome transport, pronephric duct morphogenesis, Kupffer’s vesicle development were shown for CcTrim32. Nine CcTrim members of cluster 6 were predicted to be involved in protein ubiquitination or ligase activity. Other members of this group including CcTrim13, CcTrim59, and CcTrim63 were also predicted to participate in the positive regulation of transcription, DNA-templated. Two TRIMs such as CcTrim32 and CcTrim13 showed maximum number of nine GO terms available in Table S3 of Supplemental Data Sheet 2. Regarding the cellular components category, Cytoplasm (GO:0005737) is the most enriched term, while nucleoplasm, nucleus, chromatin, and membrane component were found three times in different CcTrims. There is no specific pattern observed for each cluster; however, some members of the bottom three groups are predicted to localize in multiple sub-cellular compartments.

### 3.10. KEGG and Reactome Enrichment

**Figure 10:**
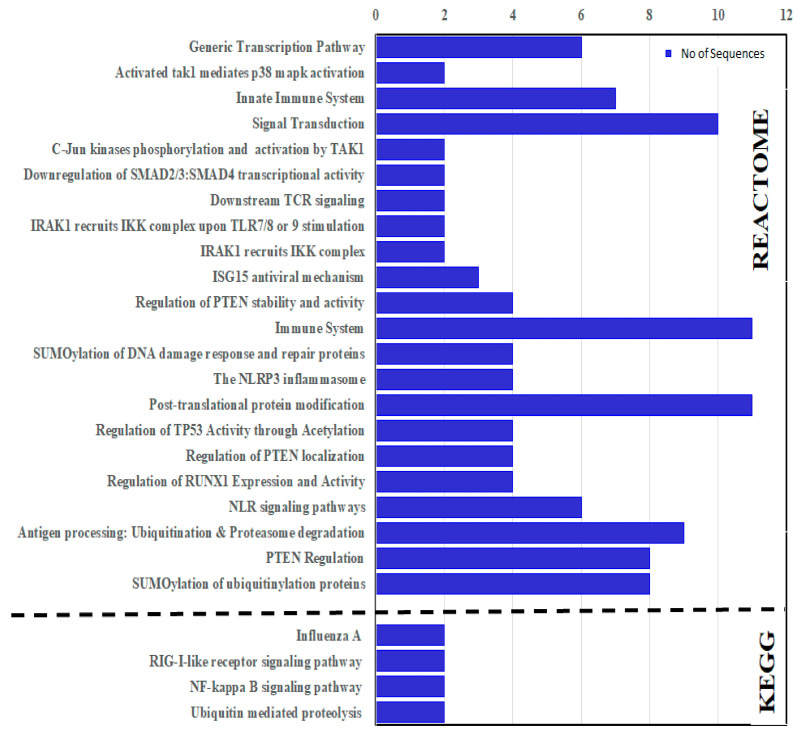
KEGG and Reactome enrichment analysis of TRIM proteins from common carp specie.

KEGG and Reactome enrichment analyses were carried out to identify the molecular and biological functions. The enrichment analyses predicted that the CcTrims genes were involved in four KEGG and 22 Reactome pathways. CcTrim25, 32, 37, and 86 are among the four genes predicted to be involved in KEGG reference pathways. Two TRIM members such as CcTrim32 and CcTrim37, have been identified as being involved in ubiquitin-mediated proteolysis. CcTrim25 and CcTrim86 members were also found to be involved in NF-kappa B signaling, RIG-I-like receptor signaling, and influenza A human infectious viral disease. Reactome pathways for common carp specie is not available. For this reason, functional enrichment of STRING network was used to identify the role of CcTrim proteins in different pathways. The Reactome results of CcTrim proteins involved in each pathway is given in Figure 10 and (Table S4 of Supplemental Data Sheet 2). Overall, thirteen TRIM proteins were involved in different pathways. Two pathways, namely the Post-translational protein modification (DRE-597592) and immune system (DRE-168256) were significantly enriched with eleven TRIM proteins. The proteins from each cluster were associated with specific pathways, such as CcTrim29, 79 and 97 of cluster 1 were predicted to be involved in eight different Reactome pathways. These include transcription related pathways, PTEN regulation, signal transduction and certain ubiquitination related pathways. TRIM proteins of cluster 2 were not associated to any Reactome pathway. Further, CcTrim25 is the only member of cluster 3, which was involved in two pathways, the immune system (DRE-168256) and ISG15 antiviral (DRE-1169408). TRIM members of cluster 4, including CcTrim35, 69, 108 and 109 were predicted to be involved in antigen processing, NLR signaling pathways, immune system, post-translation protein modification, SUMOylation and signal transduction. The members comprised of similar functional domains such as TRIMs of cluster 1 and 4 were found in four common Reactome pathways. Moreover, CcTrim33 was the only member of cluster 5 predicted to be involved in three pathways, including signal transduction, down regulation of SMAD family members transcription and generic transcription pathway. Members of cluster 6, including CcTrim13 were involved in eight pathways. Moreover, CcTrim36, 37 and 55 were associated in common pathways such as PTEN regulation and immune system. In addition, members of cluster 1 and CcTrim13 were also observed in nine common pathways.

## 4. Discussion

Common carp (*Cyprinus carpio*) is the most widely distributed freshwater fish. It has been cultivated in almost all parts of the world and is considered a very important commercial aquaculture species in South Asian and European countries. Fishery products are produced in more than 100 countries worldwide. Common carp accounts for up to 10% (over 3 million metric tons) of global annual freshwater aquaculture production [53]. The TRIM genes are well-annotated and known in other species (tetrapods and some teleosts) for their anti-viral role. Considering the importance, the TRIM genes and proteins of common carp involved in immunity are not fully understood. In this study, TRIM family of Common carp is characterized and functionally annotated which will help to determine the fundamental characteristics and will serve as preliminary evidence for future in-vitro studies. The study results will open up new avenues for future research and can be used to elucidate several mechanisms related to common carp development and immune response.

The phylogenetic analysis showed a considerable degree of conservation in the sequence identity and functional domains of TRIMs among tetrapods and teleost species. Relatively high number of proteins were observed in TRIM family. Most of the TRIMs members comprised RBCC-B30.2 domains clustered in separate groups in a phylogenetic trees. The results demonstrate that these TRIMs are closely related in all species and might perform similar type of functions. Similarly, it was observed the TRIMs that contain diverse C-terminal TRIM domains appeared as a separate monophyletic groups in phylogenetic trees. Previous reports on TRIM proteins characterization also showed the TRIM genes of primates fall into three groups [54]. In addition, the TRIM proteins of human that contain RBCC-B30.2 domains also fall into separate groups and are dissociated from TRIMs that consist discrete C-terminal domains [55]. The heatmap analysis also showed the structure and sequence identity of teleost TRIM genes is evolutionarily conserved and not very distant from each other. Moreover, the phylogenetic analysis revealed that the TRIM family of common carp also displayed the same pattern as observed in the global phylogenetic analysis. The proteins that are clustered in the same subfamily have closely similar motifs and protein architectures, suggesting the structure of the proteins was conserved among members of the same subfamily.

A total of fourteen functional domains have been identified in TRIM proteins of common carp. The order of organization in these domains was similar, but the combination of tripartite motifs was not precisely similar to tetrapods. All domains are reported in tetrapods and teleost species but some domains were not observed. For example, the transmembrane domain has been found in CcTrim 29 and 101 indicating their role as membrane integral proteins. In comparison, previous literature showed that the transmembrane domain was found in human TRIM13 and TRIM59 [6]. The presence of RING with different organization of other domains suggest different biological processes that might be specific to common carp specie. The occurrence of certain motifs across the TRIMs members implies a conserved nature of evolution in these members. The protein sequence motifs were mostly found within the domains that are conserved such as RING, B-box, coiled-coil, BBC and PRY-SPRY. Research related to protein-protein interactions on these TRIMs is still required, which will help to identify the exact function of these motifs. The CcTrim gene structures also showed a substantial difference in the number and length of intron-exon. The intron-exon diversification and uneven distribution of CcTrim genes on chromosomes also suggest duplication in the TRIM gene family. Previous studies also demonstrated that the genome of common carp undergoes an extra round of genome duplication [56]. Thus, a large number of genes are recognized in TRIM family of teleost species. The maximum number of exons and introns also suggests complex alternative splicing, consequently generating more splice variants that drive more diverse roles in cellular processes. The number of introns-exons suggest complex evolutionary mechanisms underlying the genesis of CcTrim family.

Although all proteins belong to the TRIM family but physico-chemical properties showed a significant difference in sub-family members. For instance, CcTrim44 showed a molecular weight of 36.04 KDa and the largest was CcTrim66 with a molecular weight 137.36 KDa. Similarly, the lowest PI predicted was 4.63 for CcTrim101 and 8.86 for CcTrim44. Our result indicates 62% of CcTrim proteins are acidic and tend to be positively-charged, these proteins might show a great ability to bind negative charge compounds like RNA and DNA or negatively charge proteins. The same results were also reported for some TRIMs of mammals that have the ability to bind RNA. For instance, TRIM25 and 65 have been implicated in binding to RNA for regulating RNA metabolism [57,58]. The instability index measures the stability of protein in a test tube and a value >40 is indicative of higher stability. The instability index of CcTrims reveals a range of 35.44 to 98.01 and only four proteins are predicted to be stable. The GRAVY of all proteins is negative, which indicates TRIMs of common carp are non-polar and hydrophilic. However, recent studies reported the interface of two monomers of TRIMs is mostly hydrophobic, but the side chains are hydrophilic [46]. Roughly 25% polar residues are found in core positions of TRIMs with some variation across the structure that helps stabilize the overall structure and interactions with other proteins [59].

The aligned amino acid sequence of the RING domain showed C3HC4 conserved motif in teleost species. A recent report on the comparison of MUL1-RING domain with other E3-RING domains also revealed a conserved C3HC4 motif and displayed a canonical zinc finger in a cross-brace manner to coordinate zinc ions [60]. The number of cysteines is different among B-box domains of teleost. Common carp and rainbow trout contain five cysteines, while zebrafish and channel catfish depicted four cysteine as shown in Figure 6. Previously, the logo sequence of B-box reported for invertebrates also showed five cysteine and four histidine residues [61]. Conversely, the histidine residues displayed by B-box logo sequence alignment are similar. These analyses indicated that the amino acid composition in B-box domains slightly differs among teleost, which might bring new functions specific to each organism. In the current study, the best possible tertiary structures were also modeled using the Robetta web server tool. The tertiary structures of teleost TRIM proteins show two distinct coiled-coil segments separated by non-coil segments. The functional domains were positioned sideways, thus increasing the radius for domains to interact with their ligands. The tertiary conformation is suitable for interactions, ubiquitination and substrate binding. Likewise, crystallographic and biochemical analyses of mammalian TRIMs also showed an antiparallel arrangement of coiled-coil segments, and the functional domains are positioned at opposite ends thus intensifying the formation of longer complexes [62]. Moreover, the secondary structure is comprised of the *α*-helix, *β*-sheets, and a coiled-coil region. The modeled secondary and tertiary structures of TRIMs will give a huge hint to understanding the protein structural work, interactions and drug discovery.

STRING Protein-protein interactions of CcTrim are exhibited to interact with ubiquitination associated proteins that mediate proteasomal degradation. TRIM family members, including TRIM25, 21, 8, and 11 have been shown in previous literature to mediate ubiquitylation events and degrade or modify a number of substrates [57,63,64,65]. Moreover, the STRING network shows that the TRIMs interact to interferon-induced proteins that are involved in triggering the immune response. Several reports have been described that the TRIM proteins mediate and control the up-regulation or down-regulation of interferon upon viral infection [12,66,67]. These results suggest the TRIMs of teleost also maintain the homeostasis of immune system. Moreover, the CcTrims also interact to the SMAD family proteins that participate in transforming growth factor-β(TGF*β*) pathway. To date several TRIMs including TRIM11, 25 47, 59 and 66 have also been reported to interact with a TGF*β* signaling pathway [68].

The identified TRIM proteins of common carp were subjected to functional categorization in universal Gene Ontology (GO) annotated terms. Majority of TRIMs from group 1, 2, 3 and 4 contain RBCC-B30.2 domains that were mostly associated with zinc ion binding, ligase activity, and ubiquitin-protein transferase activity in molecular function category, while in biological process protein ubiquitination was enriched. The annotation of molecular function and biological process almost complement each other. A previous study on FTR83 a member of finTRIM comprised of RBCC-B30.2 domains also revealed the modulation of signaling pathways via ubiquitination [69]. In regard to zinc ion binding annotation of CcTrims, the RING and B-box domains of TRIMs are actually a zinc finger domain that stabilize the tertiary structure of these proteins [17]. Besides zinc ion binding, ligase and ubiquitination activity, the TRIMs of groups 5 and 6 were linked to more diverse functional annotation. Moreover, CcTrim36 is associated with distinct functions such as cell cycle regulation and components of microtubule. Related studies on TRIM36 homolog of mammal also showed ubiquitin ligase activity and interaction with centromere protein-H, one of the kinetochore proteins that colocalize with α-tubulin [70]. As evident from Supplemental Data Sheet 2, some GO terms in particular positive regulation of transcription were annotated by CcTrim13, 59 and 63 that belong to same cluster. Previously, TRIM59 has been reported to play a dual role in both the transcription and ubiquitination of Beclin-1 protein (BECN1) by modulating the NF-KB pathway while it also demonstrates K-48 ubiquitination of TRAF6 for proteasomal degradation [71]. The Gene Ontology of CcTrims showed more diverse localization in a cell including cytoplasm, nucleus, and other cellular compartments given in (Supplemental Data 2, Table S3). These outcomes are consistent with previous studies reporting the localization of TRIM proteins in particular cell compartments such as TRIM2 and TRIM29 in the cytoplasm, TRIM9, 5, and 4 in cytoplasmic bodies [72].

According to pathway enrichment analyses of KEGG and Reactome the CcTrim genes are involved in multiple pathways. The KEGG pathways are strong and reliable in physical or functional relationships between genes, thus KEGG reference pathways are less in number compared to Reactome. CcTrim25 and 86 were predicted to be involved in NF-KB, RIG1, and influenza viral infection disease pathways. In comparison to KEGG analysis, several studies on TRIM proteins have been reported that showed the role of these proteins in RIG-1 like receptors, NF-KB pathway and influenza A viral disease [1]. Recent studies on TRIM25 from teleost species of zebrafish and common carp demonstrate K63-linked ubiquitination of RIG-I and positively regulates RLR signaling pathway [13,73]. Similarly, TRIM25 induced NK-KB pathway through K63-linked ubiquitination of TRAF2 [74]. These results show that the TRIM proteins of common carp also play a vital role in pathways that sense incoming viruses and inflammation. It has been well studied that TRIM proteins are involved in multitude cellular processes mediated by ubiquitination and SUMOylation. The ubiquitination leads to modulation of different cellular activities and pathways. Among Reactome, the CcTrim proteins were also significantly enriched in the pathways associated to ubiquitination of proteins, proteasomal modification and immune system. The most important and significant enriched pathways are briefly interpreted and the detailed results are provided in the Supplemental Data 2, Table S4. From Reactome results, we can easily speculate the CcTrims mainly target the mediators of immune system particular innate immunity, NLR signaling pathway, NLRP3 inflammasome and ISG15 antiviral mechanisms. The annotation of ubiquitin mediated proteolysis is also shown by KEGG, which clearly manifests the major function of these TRIMs. In comparison, previous studies have shown that TRIM65 promotes k-63 linked polyubiquitination of MDA5 helicase domain [75] and TRIM44 interacts with MAVs for k-48 linked polyubiquitination [76], consequently leading to the proteosomal degradation to trigger the immune response. These results display TRIM proteins of teleost and tetrapods are extensively involved in these pathways and modulate different substrates for degradation and post transnational modifications.

TRIM proteins not only act as E3 ligase but also exhibit SUMOylation ligase activity that governs different cellular processes. For example, TRIM19 performed SUMOylation ligase activity in the nucleus [77]. Moreover, misfolded protein degradation undergo sequential SUMOylation and ubiquitination mediated by several TRIM members, for example TRIM19 that contains RBCC structure which is in line with our findings. The CcTrim35, 79, 97, 108 and 109 contains RBCC domain (Figure 4) involved in SUMOylation of ubiquitylation proteins. Other TRIM proteins such as TRIM5, TRIM27 and TRIM36 [78] of tetrapods also contribute in SUMOylation. The other most important function shown by TRIM is regulation of TP53 acetylation which is known as the crucial gatekeeper in carcinogenesis. In comparison, previous literature also showed TRIM25 restricts the p53 function by inhibiting the acetylation required for p53 target gene transcriptional activation [79]. TRIM proteins of common carp may have multiple function, which coordinate to regulate diverse cellular processes including immune response. Considering the multiple functions exhibited by teleost TRIMs, it seems clear that TRIM family evolved as regulators, and also to eliminate infection and inflammation. The conservation of TRIM protein between teleost and mammals strongly suggests that the function and molecular mechanisms could be identical. On the other hand, a high degree of inconsistency is observed in gene and protein annotation of teleost TRIMs. In the post-genomic and next-generation sequencing era, proper nomenclature and curation of teleost gene families are deemed required. The analysis of gene ontology, protein-protein interactions, Reactome and KEGG pathway enrichment of TRIMs in common carp will provide functional resources. These analysis further need experimental validation.

## 5. Conclusions

In the present study, genome-wide analysis of the fascinating TRIM family was examined in the common carp. These genes have diverse intron-exon, motif distribution, and functional domains revealing diverse cellular functions. The phylogeny and pairwise distances of the TRIMs showed the sequences are not very distant and are conserved across vertebrates. The results of domain architecture and motifs are compatible with phylogenetic classification of proteins that are clustered into six groups (1–6). The wide range of physico-chemical parameters, uneven distribution of genes and slightly different 3D structures further revealed the diverse nature. TRIM proteins of common carp are associated to ubiquitination of immune response proteins, GTPase activity, transcription regulation and cell cycle related proteins. The detailed bioinformatic analyses and functional resources will provide a valuable foundation for future biochemical and immunological studies on teleost TRIMs.

## Figures and Tables

**Table 1:** Shows the domains that correspond to identified motifs.

S.NO	Domain/s	Corresponding Motifs
1	RING	Motif1, Motif5 partially, Motif13
2	B-box	Motif2, Motif3, Motif20
3	COILED-COIL	Motif4, Motif10
4	BBC	Motif17
5	PRY	Motif12
6	SPRY	Motif7, Motif14, Motif15, Motif19
7	PRY-SPRY	Motif8
8	B-box and COILED-COIL	Motif6
9	Not part of any domain	Motif9, 11, 16, 18

**Table 2:** Physico-chemical properties and subcellular localization of TRIM proteins in common carp. Detailed information on size of amino acids (aa), molecular weight in kilodalton (MW kDa), iso-electric point (PI), instability index, aliphatic index, and grand average of hydropathicity (GRAVY) predicted values are listed.

S.NO	Name	Subcellular Localization	Size (aa)	MW (KDA)	PI	Instability Index	Aliphatic Index	GRAVY
1	CcTrim1	Cytoplasm	538	61.6	6.56	54.94/unstable	78.23	−0.415
2	CcTrim2	Cytoplasm	669	71.39	5.90	38.61/Stable	81.90	−0.345
3	CcTrim3	Cytoplasm	772	84.88	7.54	44.31/unstable	79.78	−0.366
4	CcTrim8	Nucleus	566	63.28	7.32	63.72/unstable	69.89	−0.561
5	CcTrim9	Cytoplasm	686	76.43	6.99	38.08/stable	77.14	−0.332
6	CcTrim11	Cytoplasm	383	43.77	7.07	51.72/unstable	71.67	−0.484
7	CcTrim13	Nucleus	426	48.87	6.00	46.64/unstable	91.31	−0.118
8	CcTrim14	Cytoplasm	405	46.03	8.86	64.91/unstable	76.59	−0.647
9	CcTrim16	Cytoplasm	573	65.46	5.38	57.12/unstable	74.15	−0.610
10	CcTrim21	Cytoplasm	529	61.82	7.66	56.57/unstable	76.03	−0.640
11	CcTrim23	Cytoplasm	653	72.86	6.42	56.27/unstable	88.39	−0.183
12	CcTrim25	Nucleus	682	77.88	8.48	44.94/unstable	79.49	−0.409
13	CcTrim29	Cytoplasm	480	55.55	6.56	57.21/unstable	75.52	−0.542
14	CcTrim32	Cytoplasm	661	72.72	6.61	45.86/unstable	89.62	−0.195
15	CcTrim33	Nucleus	1065	117.34	6.16	57.40/unstable	69.48	−0.518
16	CcTrim35	Nucleus	520	58.83	8.44	46.23/unstable	81.17	−0.326
17	CcTrim36	Nucleus	744	83.17	6.45	56.89/unstable	73.87	−0.383
18	CcTrim37	Nucleus	832	92.43	5.56	52.83/unstable	76.30	−0.497
19	CcTrim39	Cytoplasm	411	47.35	5.56	59.32/unstable	73.04	−0.745
20	CcTrim44	Cytoplasm	319	36.04	4.67	53.90/unstable	70.69	−0.768
21	CcTrim45	Nucleus	577	63.92	7.24	49.88/unstable	77.83	−0.295
22	CcTrim46	Nucleus	777	86.7	7.26	51.90/unstable	81.47	−0.268
23	CcTrim47	Cytoplasm	560	63.15	7.01	46.34/unstable	74.46	−0.552
24	CcTrim54	Cytoplasm	358	40.02	5.34	48.24/unstable	79.78	−0.505
25	CcTrim55	Nucleus	521	57.26	4.75	56.06/unstable	68.87	−0.657
26	CcTrim56	Cytoplasm	400	46	8.60	62.16/unstable	70.15	−0.713
27	CcTrim58	Cytoplasm	568	65.9	5.90	46.84/unstable	79.28	−0.437
28	CcTrim59	Cytoplasm	403	45.79	5.84	47.84/unstable	98.01	−0.133
29	CcTrim62	Cytoplasm	490	56.89	6.4	48.57/unstable	81.78	−0.456
30	CcTrim63	Cytoplasm	348	39.04	4.77	50.11/unstable	79.83	−0.578
31	CcTrim65	Cytoplasm	515	58.16	6.16	47.02/unstable	74.93	−0.485
32	CcTrim66	Nucleus	1229	137.36	5.67	66.66/unstable	72.77	−0.558
33	CcTrim69	Cytoplasm	502	57.26	8.54	46.16/unstable	81.39	−0.456
34	CcTrim75	Cytoplasm	532	61.57	5.43	48.27/unstable	72.48	−0.686
35	CcTrim79	Cytoplasm	518	57.11	5.98	45.27/unstable	82.34	−0.442
36	CcTrim82	Cytoplasm	573	65.26	8.18	35.44/stable	72.34	−0.518
37	CcTrim83	Cytoplasm	415	47.45	8.33	58.08/unstable	66.99	−0.78
38	CcTrim86	Cytoplasm	603	67.73	6.41	55.82/unstable	76.2	−0.436
39	CcTrim87	Cytoplasm	384	44.57	7.98	49.40/unstable	74.87	−0.683
40	CcTrim97	Nucleus	610	67.32	6.34	39.76/stable	83.95	−0.403
41	CcTrim101	Cytoplasm	425	48.93	4.63	61.35/unstable	86.40	−0.400
42	CcTrim108	Cytoplasm	501	57.33	6.56	44.94/unstable	73.53	−0.609
43	CcTrim109	Cytoplasm	496	57.93	6.22	49.06/unstable	74.84	−0.643
44	CcTrim110	Cytoplasm	534	61.83	6.49	57.10/unstable	77.75	−0.392

## Data Availability

The datasets supporting the results of this article are included within the article and additional Supplemental Materials.

## Supplementary Materials

The following supporting information can be downloaded at: https://www.mdpi.com/article/10.3390/genes14030667/s1, Figure S1: 3D Modeled Figures of TRIM proteins in Common Carp (CcTrims); Table S1: Retrieved TRIM family members of Common Carp from NCBI; Table S2: Discovered 20 Motifs of TRIM proteins in Common Carp; Table S3: 3D modeled quality assessment of TRIM proteins in Common Carp; Table S4: Percentage proportion of secondary structural elements of TRIM proteins in Common Carp; Table S5: Distribution of CcTrim genes on Chromosome of homolog A and homolog B. www.mdpi.com/xxx/supplemental_data_sheet_1, www.mdpi.com/xxx/supplemental_data_sheet_2, Table S2_1: STRING network analysis of Common Carp TRIM; Table S2_2: Predicted functional partners of String network; Table S2_3: Gene Ontology of Common Carp TRIM proteins; Table S2_4: Reactome enrichment analysis of Common Carp TRIM proteins.
