# Supplementary material for: Genomic Analysis, Evolution and Characterization of E3 Ubiquitin Protein Ligase (TRIM) Gene Family in Common Carp (Cyprinus carpio)
Source: Genes (Basel). 2023 Mar 7;14(3):667. doi: 10.3390/genes14030667 (PMC10048487; doi:10.3390/genes14030667)
Supplement: Supplementary file 1 [file genes-14-00667-s001.zip › Supplemental_Materials_S1.pdf]

Supplemental Table S1. Retrieved TRIM family members of Common Carp from NCBI

S.NO	Gene (Rename)	Gene ID	Gene symbol	Transcript ID	Chr. No	Location start-end
1	*CcTrim1*	109067700	ftr01	XM_019084616.2	B2	27150898..27157390
2	*CcTrim2*	109085054	LOC109085054	XM_042733096.1	A1	7533611..7545195
3	*CcTrim3*	109104107	LOC109104107	XM_042771401.1	A15	19360630..19381652
4	*CcTrim8*	109104107	LOC109104107	XM_019098039.2	B13	13202457..13218813
5	*CcTrim9*	109068709	LOC109068709	XM_042777396.1	A20	3983699..4011076
6	*CcTrim11*	109051742	LOC109051742	XM_042763157.1	A9	2234991..2239668
7	*CcTrim13*	109107198	LOC109107198	XM_019120467.2	A9	16984495..16993957
8	*CcTrim14*	109112723	LOC109112723	XM_042767584.1	A12	12390296..12394485
9	*CcTrim16*	109083459	trim16	XM_042736023.1	B12	24326892..24335615
10	*CcTrim21*	109063905	LOC109063905	XM_019080913.2	B23	22305870..22313175
11	*CcTrim23*	109097861	LOC109097861	XM_042732214.1	B10	5737588..5744197
12	*CcTrim25*	109092324	trim25	XM_019106085.2	B6	10977230..10988557
13	*CcTrim29*	109089850	LOC109089850	XM_042769487.1	A13	25260525..25276843
14	*CcTrim32*	109056155	LOC109056155	XM_019073381.2	B5	28874796..28878914
15	*CcTrim33*	109080588	LOC109080588	XM_042729378.1	B8	6526235..6550492
16	*CcTrim35*	109088962	LOC109088962	XM_042770651.1	A18	25221660..25225672
17	*CcTrim36*	109094801	LOC109094801	XM_042729155.1	B8	601245..622167
18	*CcTrim37*	109088281	trim37	XM_042732814.1	B10	15858374..15878268
19	*CcTrim39*	109072822	LOC109072822	XM_042757592.1	A6	4641323..4653188
20	*CcTrim44*	109053788	trim44	XM_042715815.1	A25	19264730..19302327
21	*CcTrim45*	109083457	trim45	XM_019098242.2	B9	19610932..19616169
22	*CcTrim46*	109109264	LOC109109264	XM_042740917.1	B16	14158125..14170954
23	*CcTrim47*	109062285	LOC109062285	XM_042719462.1	B3	1848355..1858752
24	*CcTrim54*	109053743	LOC109053743	XM_042746130.1	B20	21769937..21783032
25	*CcTrim55*	109103052	trim55b	XM_042714429.1	A24	17167297..17173238
26	*CcTrim56*	109061665	LOC109061665	XM_019078765.2	B19	4843826..4849195
27	*CcTrim58*	109068797	LOC109068797	XM_042728800.1	B7	43933707..43941261
28	*CcTrim59*	109093007	trim59	XM_042765804.1	A11	227890..230574
29	*CcTrim62*	109063795	LOC109063795	XM_042766842.1	A11	22603627..22650278
30	*CcTrim63*	109084181	trim63a	XM_042758022.1	A6	10797869..10799420,

31	*CcTrim65*	109112030	LOC109112030	XM_042736002.1	B12	23717168..23721912
32	*CcTrim66*	109052967	trim66	XM_042726823.1	B7	16531772..16549988
33	*CcTrim69*	109110299	trim69	XM_042765359.1	A10	15487874..15492537
34	*CcTrim75*	109102694	LOC109102694	XM_042713875.1	A23	21621158..21626991
35	*CcTrim79*	109113431	ftr79	XM_042777732.1	A20	14639446..14643238
36	*CcTrim82*	109090511	ftr82	XM_019104337.2	B5	8472805..8479235
37	*CcTrim83*	109093007	trim59	XM_042724100.1	B5	8484806..8491527
38	*CcTrim86*	109075219	ftr86	XM_019091148.2	B15	3790068..3797583
39	*CcTrim87*	109111766	ftr87	XM_019124764.2	A2	25052322..25058301
40	*CcTrim97*	109113434	ftr97	XM_042777733.1	A20	14644198..14650260
41	*CcTrim101*	109053909	LOC109053909	XM_042751511.1	B23	11357401..11361725
42	*CcTrim108*	109053796	LOC109053796	XM_042740855.1	B16	13387173..13390989
43	*CcTrim109*	109071509	LOC109071509	XM_042740685.1	B16	10567139..10573782
44	*CcTrim110*	109079603	trim110	XM_042777371.1	A2	19179708..19183776

Supplemental Table S2. Discovered 20 Motifs of TRIM proteins in Common Carp

**Motif 1**	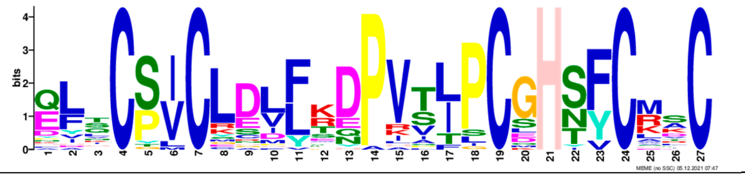
**Motif 2**	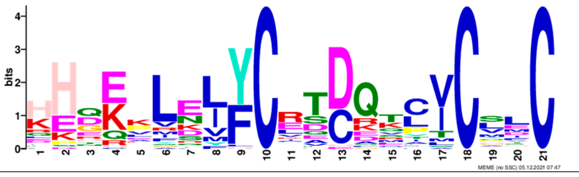
**Motif 3**	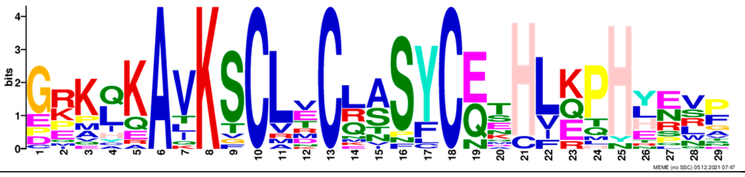
**Motif 4**	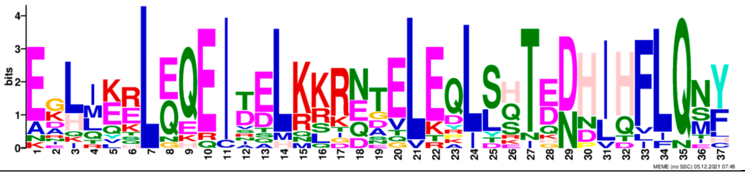

**Motif 5**	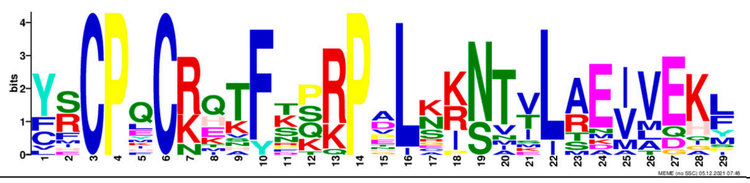
**Motif 6**	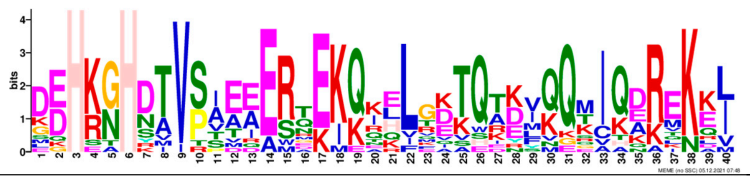
**Motif 7**	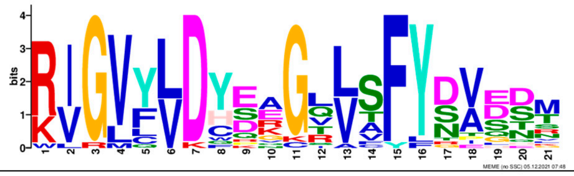
**Motif 8**	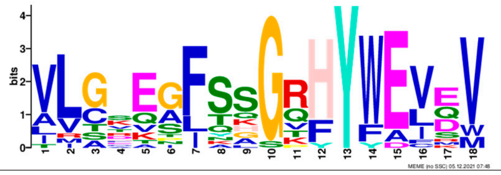
**Motif 9**	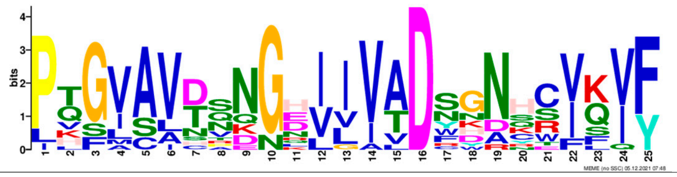
**Motif 10**	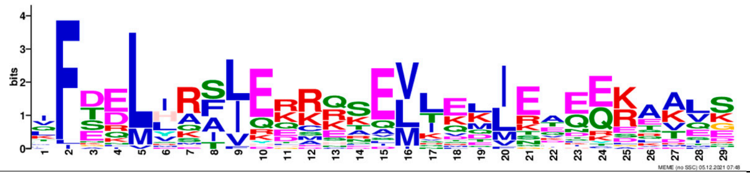
**Motif 11**	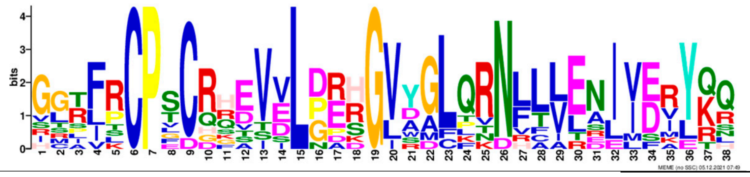
**Motif 12**	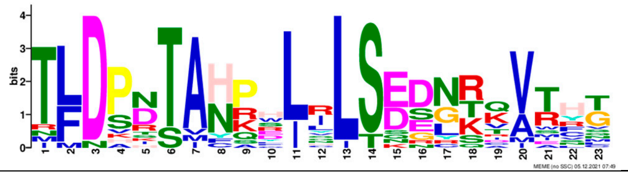

**Motif 13**	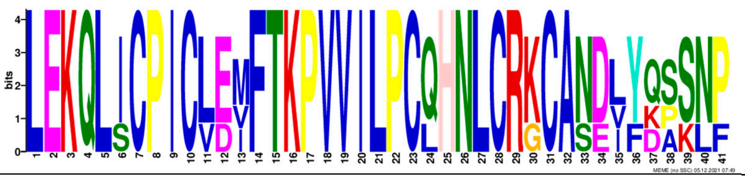
**Motif 14**	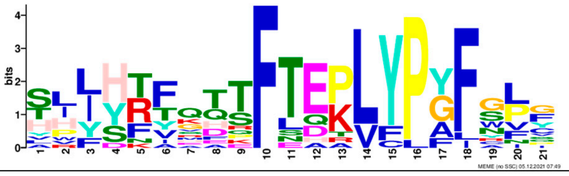
**Motif 15**	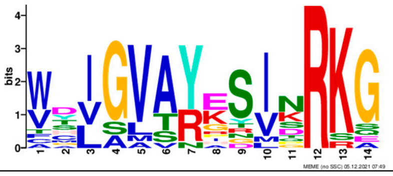
**Motif 16**	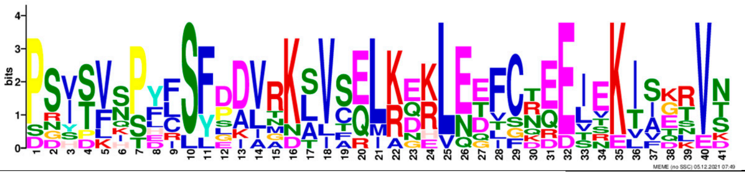
**Motif 17**	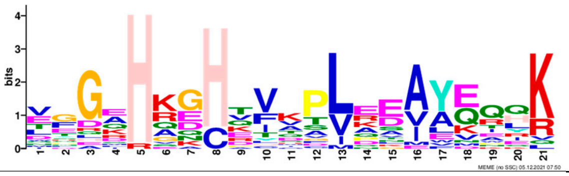
**Motif 18**	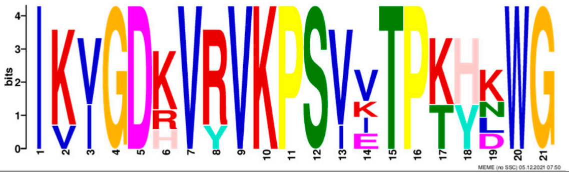
**Motif 19**	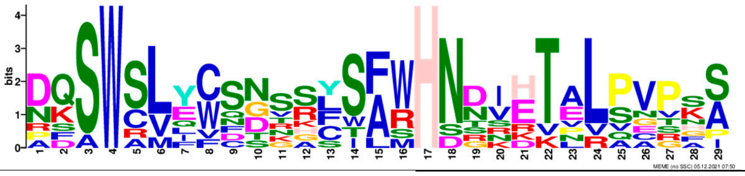
**Motif 20**	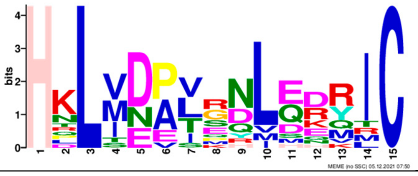

Figure S1. 3D Modeled Figures of TRIM proteins in Common Carp (CcTrims)

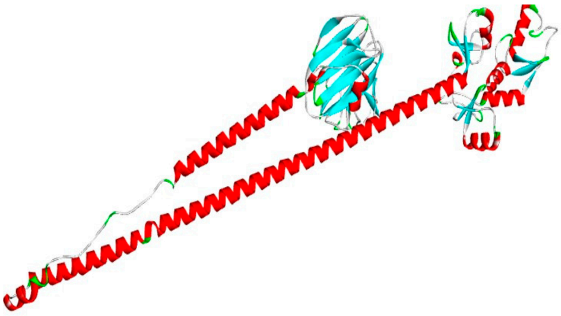

Figure1. 3D model of CcTrim1

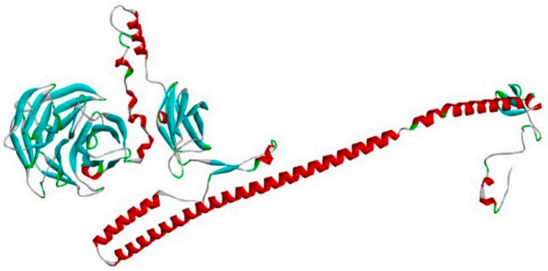

Figure2. 3D model of CcTrim2

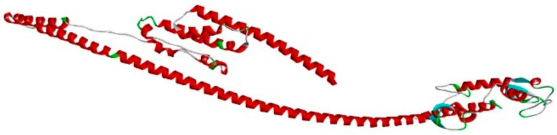

Figure3. 3D model of CcTrim3

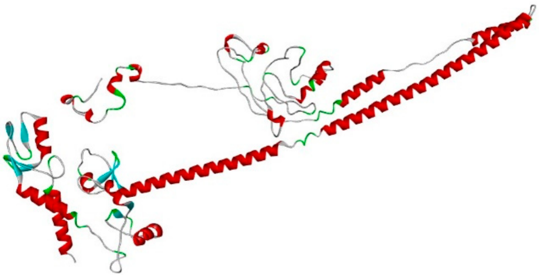

Figure4. 3D model of CcTrim8

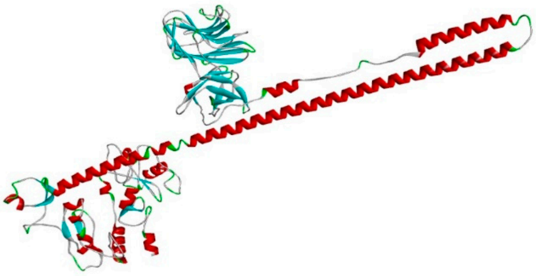

Figure5. 3D model of CcTrim9

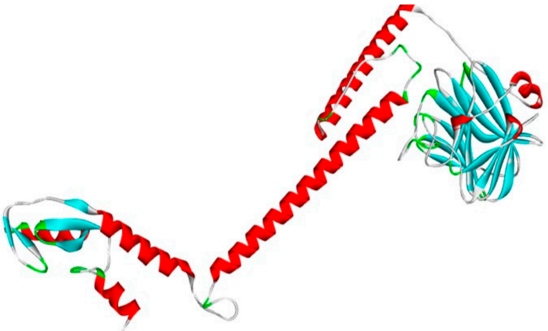

Figure6. 3D model of CcTrim11

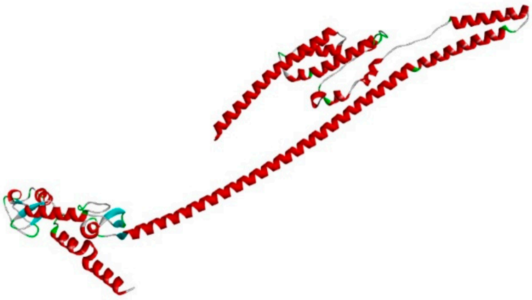

Figure7. 3D model of CcTrim13

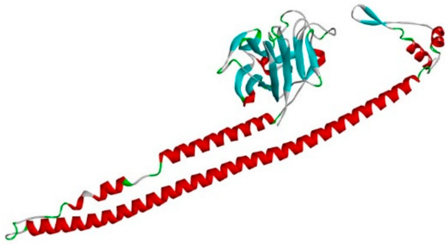

Figure8. 3D model of CcTrim14

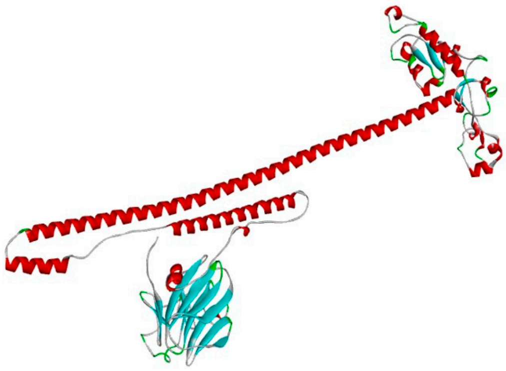

Figure9. 3D model of CcTrim16

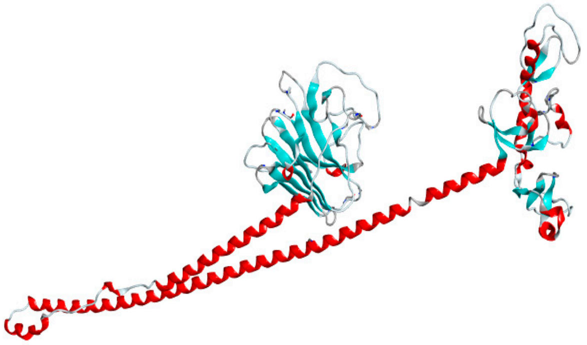

Figure10. 3D model of CcTrim21

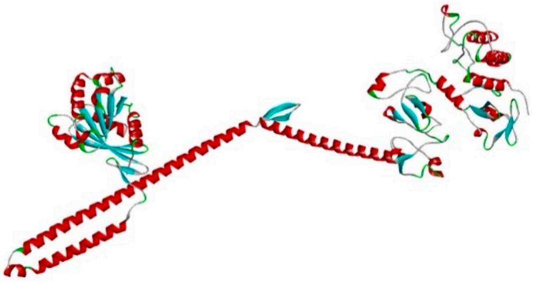

Figure11. 3D model of CcTrim23

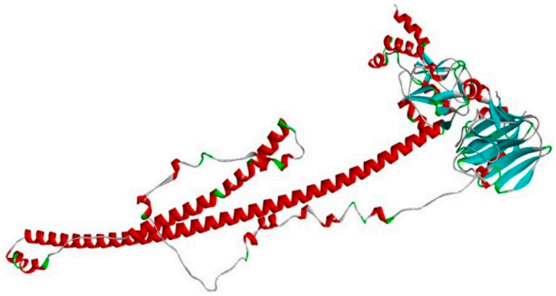

Figure12. 3D model of CcTrim25

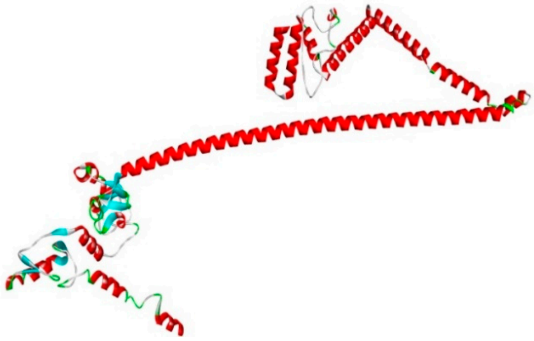

Figure13. 3D model of CcTrim29

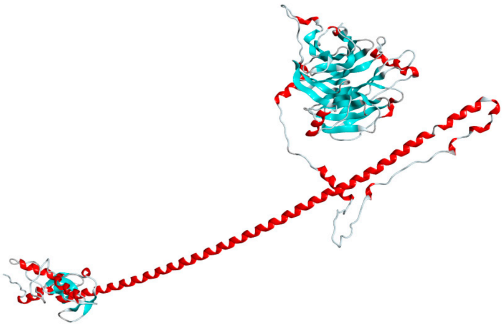

Figure14. 3D model of CcTrim32

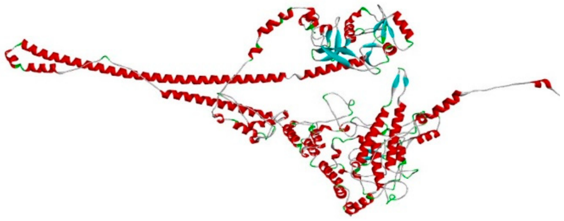

Figure15. 3D model of CcTrim33

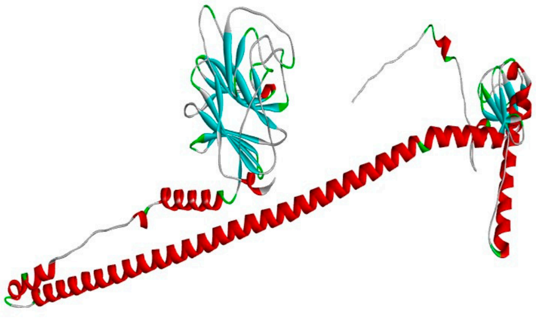

Figure16. 3D model of CcTrim35

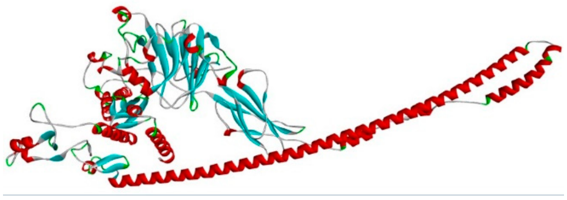

Figure17. 3D model of CcTrim36

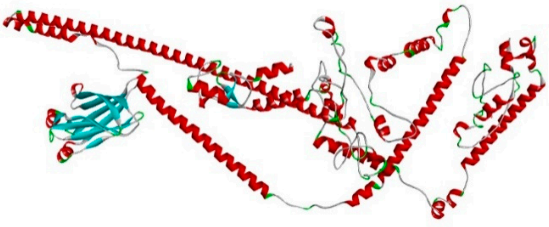

Figure18. 3D model of CcTrim37

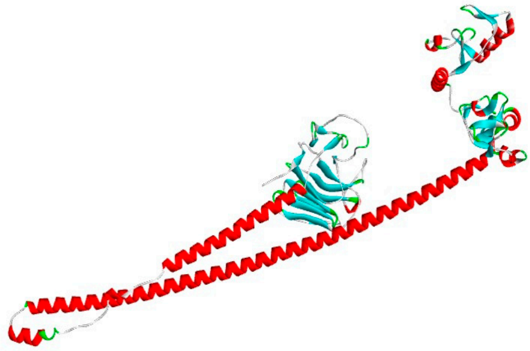

Figure19. 3D model of CcTrim39

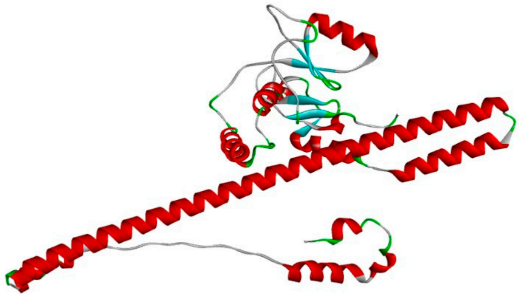

Figure20. 3D model of CcTrim44

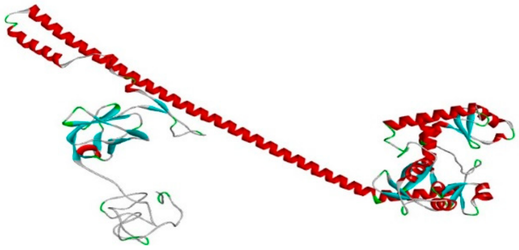

Figure21. 3D model of CcTrim45

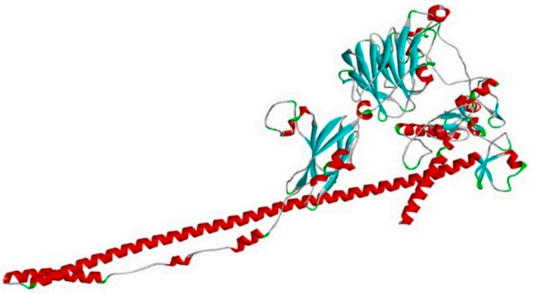

Figure22. 3D model of CcTrim46

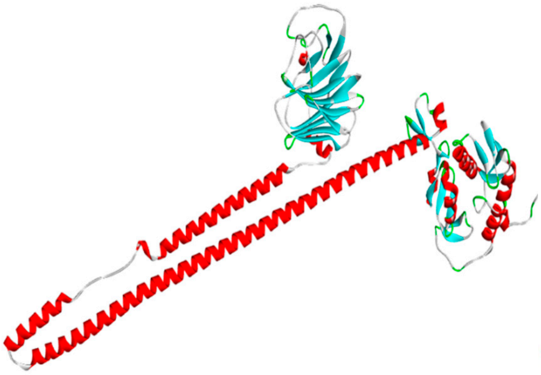

Figure23. 3D model of CcTrim47

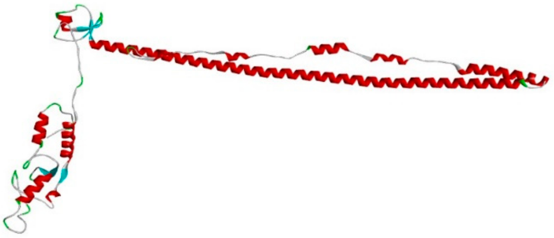

Figure24. 3D model of CcTrim54

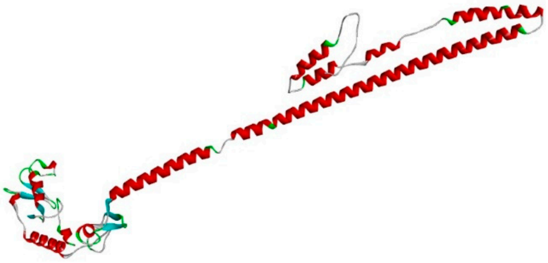

Figure25. 3D model of CcTrim55

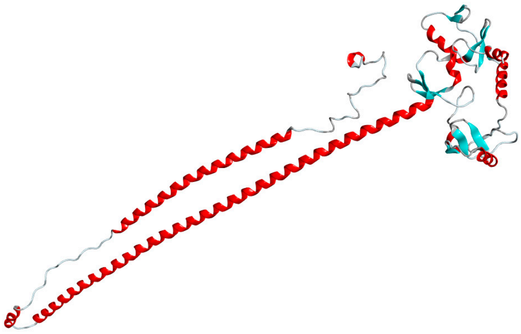

Figure26. 3D model of CcTrim56

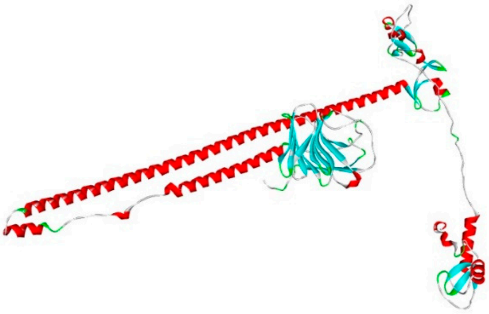

Figure27. 3D model of CcTrim58

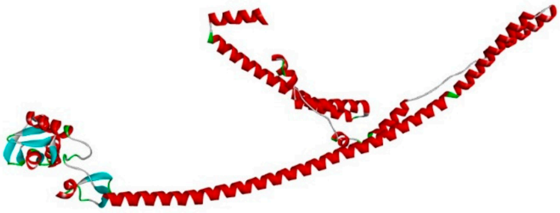

Figure28. 3D model of CcTrim59

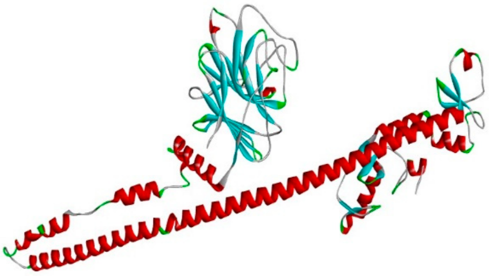

Figure29. 3D model of CcTrim62

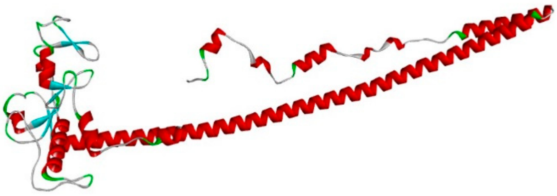

Figure30. 3D model of CcTrim63

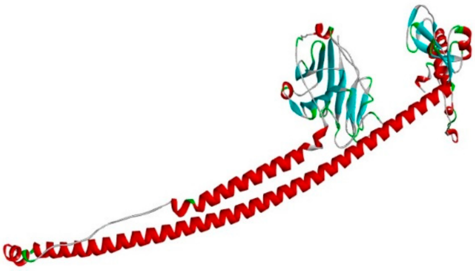

Figure31. 3D model of CcTrim62

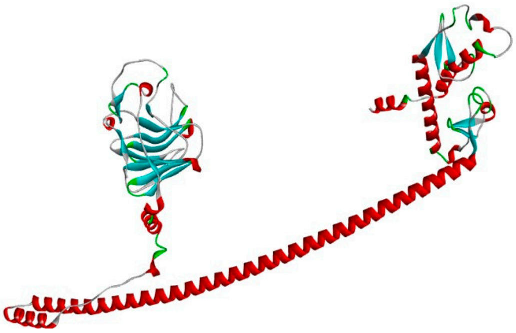

Figure32. 3D model of CcTrim63

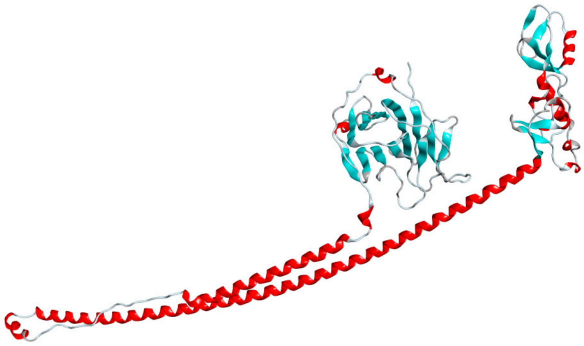

Figure33. 3D model of CcTrim65

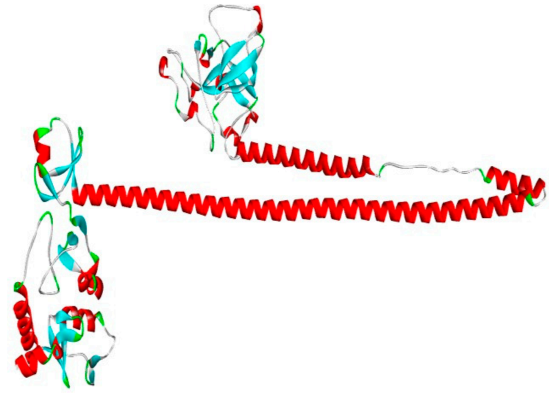

Figure34. 3D model of CcTrim69

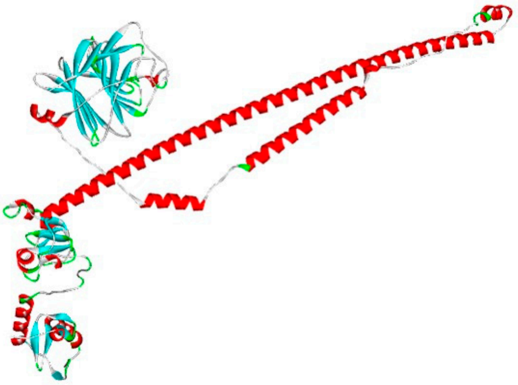

Figure35. 3D model of CcTrim75

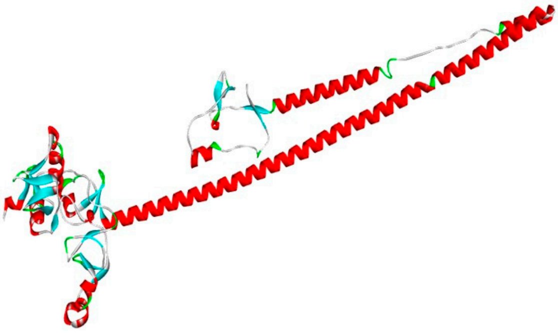

Figure36. 3D model of CcTrim79

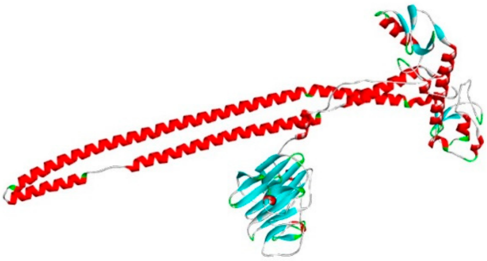

Figure37. 3D model of CcTrim82

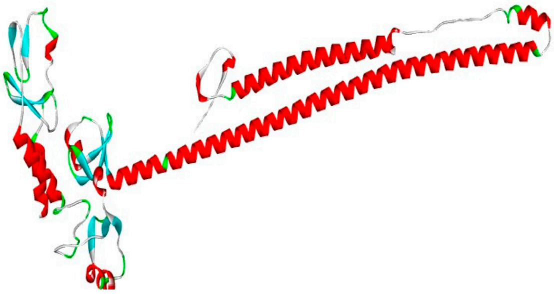

Figure38. 3D model of CcTrim83

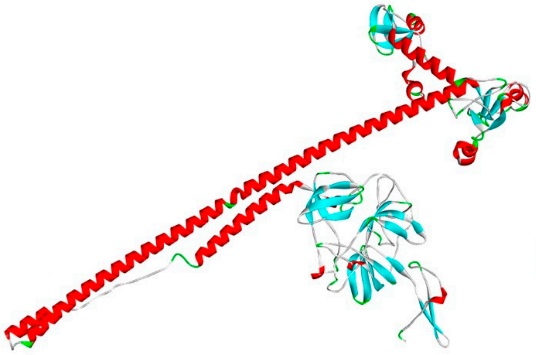

Figure39. 3D model of CcTrim97

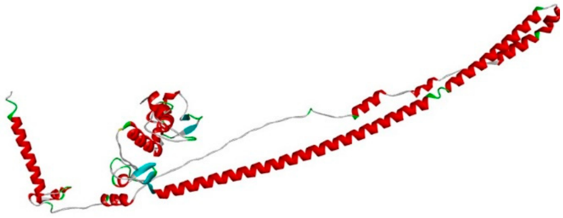

Figure40. 3D model of CcTrim101

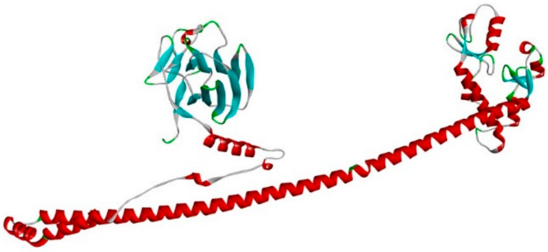

Figure41. 3D model of CcTrim108

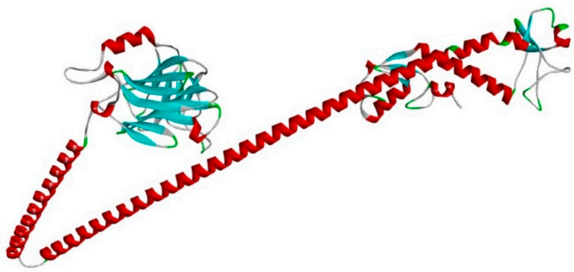

Figure42. 3D model of CcTrim109

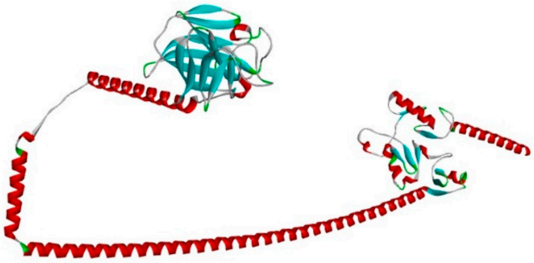

Figure43. 3D model of CcTrim110

Supplemental Table S3. 3D modeled quality assessment of TRIM proteins in Common Carp.

CcTrim Member	RAMACHANDRAN PLOT STATISTICS (%)				QMEAN4	ERRAT QUALITY FACTOR	VERIFY 3-D
	Most favoured regions	Additional allowed regions	Generously allowed regions	Disallowed regions			
CcTrim1	91.7%	6.9%	0.6%	0.8%	1.34	93.3837	62.08%
CcTrim2	88.4%	10.1%	1.0%	0.5%	1.95	96.1774	72.20%
CcTrim3	91.9%	6.8%	0.0%	1.3%	0.33	99.2823	54.93%
CcTrim8	77%	18.7%	2.0%	2.4%	-1.57	87.0674	54.06%
CcTrim9	85.9%	10.7%	1.8%	1.6%	0.20	82.8313	70.12%
CcTrim11	91.1%	7.8%	0.6%	0.6%	1.52	84.5109	65.80%
CcTrim13	91.8%	6.8%	0.0%	1.3%	0.33	99.2823	54.93%
CcTrim14	87.8%	9.5%	1.4%	1.4%	1.61	92.8571	58.52%
CcTrim16	88.9%	9.4%	0.6%	1.1%	0.78	91.9414	66.67%
CcTrim21	91.6%	6.8%	0.6%	1.0%	2.20	91.3295	73.16%
CcTrim23	89.5%	8.1%	1.2%	1.2%	0.67	94.6541	63.40%
CcTrim25	89.2%	9.1%	1.2%	1.2%	0.76	92.4012	53.23%
CcTrim29	89.4%	8.4%	1.3%	0.9%8	0.09	99.1525	43.13%
CcTrim32	84.8%	13.5%	0.9%	0.9%	0.18	92.79	63.09%
CcTrim33	86.7%	11.3%	1.0%	1.1%	-1.21	92.9064	48.54%
CcTrim35	89.7%	9.2%	0.4%	0.6%	2.40	93.8614	64.81%
CcTrim36	88.0%	9.6%	1.1%	1.4%	0.75	92.7989	71.10%
CcTrim37	86.7%	10.6%	1.6%	1.1%	-0.45	91.5528	52.04%
CcTrim39	88.7%	9.7%	0.2%	1.4%	2.00	92.381	68.73%
CcTrim44	91.3%	7.7%	0.3%	0.7%	0.71	100	58.62%
CcTrim45	87.5%	8.6%	1.6%	2.4%	-0.33	93.0147	66.67%

CcTrim46	88.0%	9.2%	1.3%	1.5%	0.26	85.9788	60.36%
CcTrim47	91.9%	6.4%	1.0%	0.8%	1.55	94.0109	65.71%
CcTrim54	87.8%	10.9%	0.9%	0.8%	0.58	99.1202	44.41%
CcTrim55	90.6%	7.1%	0.6%	1.6%	0.29	97.619	44.93%
CcTrim56	92.1%	7.4%	0.3%	0.3%	1.25	95.9184	45.50%
CcTrim58	91.4%	6.9%	0.8%	1.0%	1.42	90.146	55.28%
CcTrim59	93.3%	5.6%	0.3%	0.8%	1.37	97.4684	53.35%
CcTrim62	88.7%	9.0%	1.1%	1.1%	1.08	93.5417	76.53%
CcTrim63	91.2%	8.2%	0.2%	0.2%	1.24	97.6401	49.43%
CcTrim65	91.0%	7.3%	0.6%	1.1%	1.13	93.0966	60.58%
CcTrim66	88.5%	10.9%	0.6%	0.0%	-3.46	78.2353	72.04%
CcTrim69	90.3%	7.3%	0.0%	2.4%	2.05	92.623	56.57%
CcTrim75	90.6%	8.5%	0.4%	0.4%	2.35	96.5583	65.98%
CcTrim79	90.8%	8.3%	0.4%	0.4%	1.01	93.6884	66.02%
CcTrim82	91.0%	8.0%	0.4%	0.6%	1.08	88.551	65.62%
CcTrim83	88.2%	9.4%	0.8%	1.6%	0.27	96.9543	61.45%
CcTrim86	90.4%	8.1%	0.7%	0.7%	0.09	93.9086	59.37%
CcTrim87	90.1%	7.4%	1.1%	1.4%	0.09	95.4424	48.70%
CcTrim97	85.3%	12.2%	1.3%	1.3%	0.92	92.3208	68.20%
CcTrim101	89.3%	8.6%	0.8%	1.3%	1.10	88.8608	28.94%
CcTrim108	91.2%	7.1%	0.4%	1.3%	1.18	95.935	60.28%
CcTrim109	89.8%	8.8%	0.2%	1.1%	1.74	91.9588	66.73%
CcTrim110	91.2%	7.8%	0.2%	0.8%	1.03	90.4762	60.11%

Supplemental Table S4. Percentage proportion of secondary structural elements of TRIM proteins in Common Carp

Protein Name	Predicted % of helix element	Predicted % of beta-sheet element	Predicted % of coiled-coil element
**Cluster 1**			
CcTrim79	36	14	49
CcTrim97	33	16	51
CcTrim29	46	7	47
CcTrim82	36	15	49
CcTrim83	53	3	44
CcTrim87	53	3	44
CcTrim11	29	20	51
CcTrim56	51	3	46
CcTrim1	38	17	46
CcTrim47	34	16	50
CcTrim86	37	14	49
**Cluster 2**			
CcTrim58	37	15	48
CcTrim39	32	17	49
CcTrim21	38	14	48
CcTrim75	36	17	49
**Cluster 3**			
CcTrim65	33	19	48
CcTrim110	42	18	40
CcTrim14	33	17	50
CcTrim16	33	15	52
CcTrim25	35	12	53
**Cluster 4**			
CcTrim44	43	10	47
CcTrim35	33	19	48
CcTrim62	40	18	42
CcTrim69	38	16	46
CcTrim108	34	18	48
CcTrim109	38	17	45
**Cluster 5**			
CcTrim23	50	14	36
CcTrim32	23	27	50
CcTrim33	27	4	69
CcTrim66	20	4	76
CcTrim45	35	15	50
CcTrim2	36	21	43
CcTrim3	23	26	51
**Cluster 6**			
CcTrim13	56	5	39
CcTrim59	34	4	62
CcTrim37	34	7	59
CcTrim8	38	8	44
CcTrim36	34	17	49

CcTrim46	33	16	51
CcTrim9	24	20	56
CcTrim63	57	5	38
CcTrim101	50	4	46
CcTrim54	57	4	39
CcTrim55	55	5	40

Supplemental Table S5. Distribution of CcTrim genes on Chromosome of homolog A and homolog B

Serial NO.	Chromosome Homolog A	Chromosome Homolog B
1	CcTrim2	CcTrim8
2	CcTrim3	CcTrim16
3	CcTrim9	CcTrim21
4	CcTrim11	CcTrim23
5	CcTrim13	CcTrim25
6	CcTrim14	CcTrim32
7	CcTrim29	CcTrim33
8	CcTrim35	CcTrim36
9	CcTrim39	CcTrim37
10	CcTrim44	CcTrim45
11	CcTrim47	CcTrim46
12	CcTrim55	CcTrim47
13	CcTrim59	CcTrim54
14	CcTrim63	CcTrim56
15	CcTrim69	CcTrim58
16	CcTrim75	CcTrim62
17	CcTrim79	CcTrim65
18	CcTrim87	CcTrim66
19	CcTrim97	CcTrim82
20	CcTrim110	CcTrim83
21		CcTrim86
22		CcTrim101
23		CcTrim108
24		CcTrim109
